# RipE expression correlates with high ATP levels in *Ehrlichia*, which confers resistance during the extracellular stage to facilitate a new cycle of infection

**DOI:** 10.3389/fcimb.2024.1416577

**Published:** 2024-10-01

**Authors:** Rory C. Chien, Mingqun Lin, Nan Duan, Stephen Denton, Jeffrey Kawahara, Yasuko Rikihisa

**Affiliations:** Department of Veterinary Biosciences, The Ohio State University, Columbus, OH, United States

**Keywords:** *Ehrlichia*, RipE, bacterial ATP, genetic complementation, transposon mutant, mouse, CtrA

## Abstract

Ehrlichiosis is a potentially life-threatening disease caused by infection with the obligatory intracellular bacteria *Ehrlichia* species. *Ehrlichia japonica* infection of mice provides an animal model of ehrlichiosis as it recapitulates full-spectrum and lethal ehrlichiosis in humans. The *E. japonica* transposon mutant of *EHF0962*, which encodes a previously uncharacterized hypothetical protein, is attenuated in both infection and virulence in mice. EHF0962 was hence named here as resistance-inducing protein of *Ehrlichia* (RipE). Using this Δ*ripE* mutant, we studied how RipE protein contributes to *Ehrlichia* pathogenesis. *Ehrlichia* species have an intracellular developmental cycle and a brief extracellular stage to initiate a new cycle of infection. Majority of RipE proteins were expressed on the surface of the smaller infectious dense-core stage of bacteria. Extracellular Δ*ripE E. japonica* contained significantly less adenosine triphosphate (ATP) and lost infectivity more rapidly in culture compared with wild-type (WT) *E. japonica*. Genetic complementation in the Δ*ripE* mutant or overexpression of *ripE* in WT *E. japonica* significantly increased bacterial ATP levels, and RipE-overexpressing *E. japonica* was more virulent in mice than WT *E. japonica*. RipE is conserved among *Ehrlichia* species. Immunization of mice with recombinant RipE induced an *in vitro* infection-neutralizing antibody, significantly prolonged survival time after a lethal dose of *E. japonica* challenge, and cross-protected mice from infection by *Ehrlichia chaffeensis*, the agent of human monocytic ehrlichiosis. Our findings shed light on the extracellular stage of *Ehrlichia*, highlighting the importance of RipE and ATP levels in *Ehrlichia* for extracellular resistance and the next cycle of infection. Thus, RipE is a critical *Ehrlichia* protein for infection as such can be a potential vaccine target for ehrlichiosis.

## Introduction

The family *Anaplasmataceae* consists of a group of Gram-negative, obligate intracellular alpha-proteobacteria that cause nonspecific febrile diseases in susceptible animals and humans (Rikihisa, 1991; Dumler et al., 2001; Rikihisa, 2010). Although the diseases are usually self-limiting in humans, severe illness can occur upon infection with *Ehrlichia chaffeensis* or *Anaplasma phagocytophilum*, with some cases proven fatal (Bakken and Dumler, 2000). Notably, the Centers for Disease Control and Prevention must be notified for every infection case, which shows that disease incidence has increased significantly in the past two decades (CDC, 2024). *E. chaffeensis* causes the systemic disease human monocytic ehrlichiosis (HME), for which doxycycline is the only treatment (CDC, 2024). To facilitate current efforts to develop an HME vaccine (McGill et al., 2016; Thomas, 2016; Budachetri et al., 2020, Budachetri et al., 2022), more research is needed to understand HME pathogenesis and to discover effective therapeutic and vaccine targets.

An ehrlichial species was recently characterized by whole-genome sequencing and classified as *Ehrlichia japonica* sp. nov (Lin et al., 2021; Oren and Garrity, 2022). This species was originally isolated from *Ixodes ovatus* ticks in Japan and was previously known as an *I*. *ovatus* ehrlichia (IOE) agent or *Ehrlichia* sp. HF strain. Phylogenetic analysis revealed that *E. japonica* is closely related to *E. chaffeensis* based on 16S rRNA and GroEL protein sequences (Shibata et al., 2000). Laboratory mice challenged with *E. japonica* develop an acute systemic infection followed by death that resembles severe HME (Shibata et al., 2000; Sotomayor et al., 2001; Zhang et al., 2024). Thus, over the past several years, *E. japonica* infection in immunocompetent mice has been increasingly used as a model for studying HME (Shibata et al., 2000; Okada et al., 2001, Okada et al., 2003; Winslow et al., 2005; Ismail et al., 2006; Habib et al., 2016; Haloul et al., 2019; Ahmed and Ismail, 2020; Ismail et al., 2022; Sharma et al., 2023). As *E. japonica* causes a dose-dependent, full-spectrum disease with an LD_50_ of approximately 100 bacteria (Bekebrede et al., 2020), this mouse model provides an excellent way to investigate ehrlichiosis pathogenesis *in vivo* and to bridge the knowledge gap between *E. chaffeensis* infection and severe HME.

To overcome the mammalian immune system, establish infection, and cause disease within the host, *Ehrlichia* must utilize additional strategies, such as *in vivo* virulence factors, beyond those required for infection of eukaryotic cells in culture (Rikihisa, 2021). However, the vast majority of *Ehrlichia* virulence factors and their functions *in vivo* remain undetermined. According to recently published whole-genome sequencing data, *E. japonica* contains 866 protein-coding genes (Lin et al., 2021). Besides those genes essential for protein or nucleotide biosynthesis and key metabolic pathways, the functions of 244 proteins remain unknown and hence such proteins are annotated as hypothetical proteins. Because *Ehrlichia* spp. and other members of the family *Anaplasmataceae* have evolved to have a relatively small genome (Dunning Hotopp et al., 2006; Rikihisa, 2015), it is very likely that at least some, if not all, of these hypothetical proteins could have played key roles in the evolutionary success of *Ehrlichia* spp. To investigate the functions of these unknown *Ehrlichia* genes, we used a Himar1 transposon random mutagenesis system (Felsheim et al., 2006) to create a library of *E. japonica* that comprises at least 158 distinct mutants, carrying genetic mutations at different loci (Bekebrede et al., 2020). Among the mutants that can replicate well in cell culture but are attenuated in mouse virulence is the H59, a clone of a Himar1 insertional mutant disrupting *EHF_0962* gene (*EHF_0962*::Himar1), which caused significantly lower bacterial loads in the blood, liver, and spleen with reduced clinical signs at day 7 post-infection (pi) (Bekebrede et al., 2020). *EHF_0962* encodes a previously uncharacterized hypothetical protein with 120 amino acid residues (molecular mass, ~13.5 kDa, pI 5.78, GenBank accession no. WP_052349286.1) (Lin et al., 2021), which was named in this study as resistance-inducing protein of *Ehrlichia* (RipE). Here, we characterized RipE functions in *E. japonica* pathogenesis in mice. Our results suggest that RipE helps bacteria survival during the extracellular stage of *Ehrlichia*, thereby facilitating *in vivo* infection.

## Results

### Characteristics of the Δ*ripE* mutant and RipE

**Figure 1:**
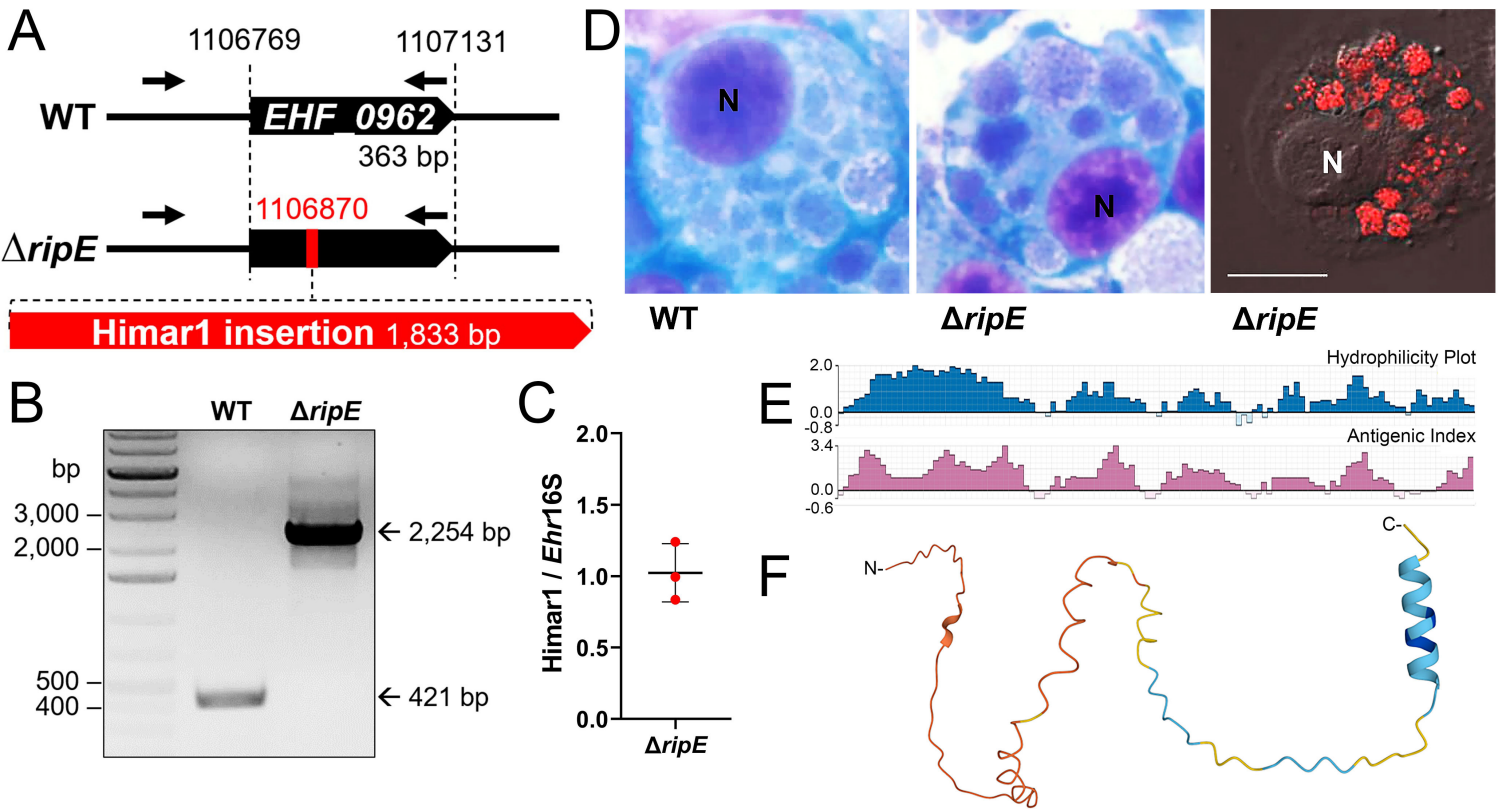
Clonality of Δ*ripE E*. *japonica* and bioinformatic analysis of RipE protein. **(A)** Diagram of *E*. *japonica* genome showing Δ*ripE* with Himar1 transposon insertion in locus *EHF_0962* (*ripE*). GenBank Reference Sequence number: NZ_CP007474.1. ORFs (black pentagon arrows). Himar1 insertion containing spectinomycin/streptomycin resistance gene and *mCherry* (red pentagon arrow). *ripE*-specific flanking PCR primers (arrows). **(B)** *ripE*-specific flanking PCR for Δ*ripE E*. *japonica* clonality verification. **(C)** Copy numbers of the mCherry gene vs. the single-copy *Ehrlichia* 16S rRNA gene in Δ*ripE E. japonica* genome determined by qPCR from three different batches of cultures. **(D)** WT and Δ*ripE E*. *japonica* cultured in DH82 cells. Note the characteristic *Ehrlichia* micro-colonies (morulae) by HEMA3 stain in the cytoplasm of infected DH82 cells, and Δ*ripE* mutants were also detected by mCherry fluorescence using a Leica Thunder fluorescence microscope. N, nucleus. Scale bar, 10 µm. **(E)** Antigenic index and hydrophilicity plot of RipE by Protean prediction. **(F)** RipE protein 3D structure prediction by AlphaFold.

To study the functions of RipE in *E. japonica* pathogenesis, we first confirmed the stable clonality of the Δ*ripE* mutant H59 (Bekebrede et al., 2020). Himar1 insertion site-specific flanking PCR for *ripE* (**Supplementary Table S1**) (Bekebrede et al., 2020) showed that the Δ*ripE* mutant produced a single, larger PCR product containing the Himar1 insert (2,254 bp) compared with WT *E. japonica* (421 bp, **Figures 1A, B**). Himar1 insertions occur mostly once and rarely twice per genome (Cartman and Minton, 2010; Cain et al., 2020); therefore, to verify that there were no additional Himar1 insertions in the Δ*ripE* mutant genome, quantitative PCR (qPCR) was performed, and the results showed a ratio of 1:1 for the copy number of the mCherry gene vs. the 16S rRNA gene, which is a single copy per *Ehrlichia* genome (**Figure 1C**). Wild-type (WT) *E. japonica* and the Δ*ripE* mutant had similar growth curves when cultured in DH82 canine macrophages or ISE6 tick cells (Bekebrede et al., 2020). The intracellular micro-colonies (morulae) formed by WT and Δ*ripE E. japonica* are essentially indistinguishable by HEMA3 staining (Bekebrede et al., 2020) (**Figure 1D**), whereas the Δ*ripE* mutant could be easily distinguished from WT *E. japonica* owing to its mCherry fluorescence under a fluorescence microscope (**Figure 1D**). To investigate whether the *ripE* gene is involved in mammalian endothelial tropism and infection, we also compared the growth curves of WT and Δ*ripE E. japonica* in the RF/6A rhesus monkey endothelial cell line, but no significant difference was observed (**Supplementary Figure S1**).

Protein Blast search revealed that RipE homologs were identified in all sequenced *Ehrlichia* spp., including *E. muris*, *E. chaffeensis*, *E. canis*, and *E. ruminantium* (**Table 1**). However, no RipE homologs could be found beyond the genus *Ehrlichia*, even in other closely related members in the family *Anaplasmataceae* such as *Anaplasma* and *Neorickettsia* spp., suggesting that RipE is a unique protein that evolved among *Ehrlichia* spp. As RipE lacks any known motifs cataloged in the NCBI database, bioinformatic analyses were performed to determine its secondary-structure characteristics. Analysis using Protean (DNASTAR Lasergene) revealed that RipE consists of six hydrophilic regions each with a high antigenic index intervened with short hydrophobic linkers (**Figure 1E**). Three-dimensional structure prediction based on AlphaFold (Jumper et al., 2021) indicated that RipE contains mostly intrinsically disordered regions, which are flexible and lack well-defined three-dimensional structures except for one α-helix at the C-terminus and a short β-sheet near the N-terminus (**Figure 1F**).

**Table 1:** Homologous proteins of *E. japonica* RipE in *Ehrlichia* species^1^.

Organism	Locus ID	Gene Product	AA	% AA Identity	Blast E-value
*E. japonica*	EHF_0962	Hypothetical protein (**RipE**)	119	-	-
*E. muris* Wisconsin	PVA18_04380	Hypothetical protein	120	65	5e-37
*E. muris* AS145	EMUR_00190	Hypothetical protein	104	63	2e-35
*E. chaffeensis*	ECH_0079	Hypothetical protein	134	46	1e-25
*E. canis*	Ecaj_0047	Hypothetical protein	133	43	3e-20
*E. ruminantium*	FDZ68_00295	Hypothetical protein	102	22	7e-04

^1^Protein homologs to *E. japonica* RipE were identified by Blastp search against the NCBI protein database NR (all non-redundant protein sequences) with default parameters. Only unique matches for each representative species were listed in the table.

*E. muris* Wisconsin, *E. muris* subsp. eauclairensis Wisconsin; *E. muris* AS145, *E. muris* subsp. muris AS145.

### RipE expression by *E. japonica* in DH82 cells

**Figure 2:**
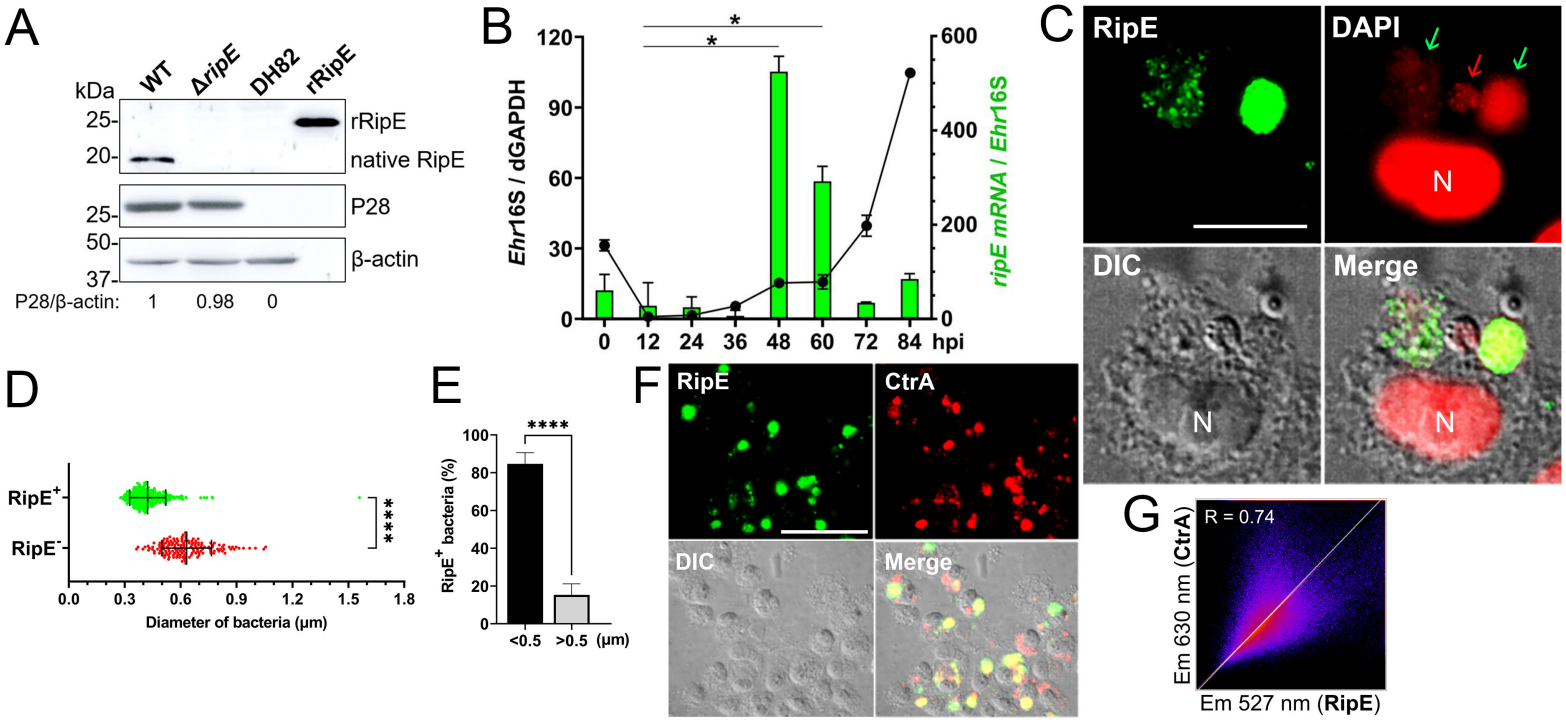
RipE is not expressed by the Δ*ripE* mutant and differentially expressed by WT *E*. *japonica.* **(A)** Lysates of WT and Δ*ripE E*. *japonica-*infected and uninfected DH82 cells and rRipE were analyzed by Western blotting using antibodies against rRipE, *Ehrlichia* P28, and β-actin. **(B)** Growth curve of *Ehrlichia* (black line) and expression of RipE mRNA (green bar) by WT *E*. *japonica* in DH82 cells were analyzed by RT-qPCR. *E*. *japonica* 16S rRNA (*Ehr*16S) expression was normalized by dog GAPDH mRNA (dGAPDH). hpi, hours post infection. RipE mRNA/*Ehr16S* with the ratio at 12 hpi set as 1. Data are displayed by the 2^−ΔΔCT^ method and shown as means ± standard deviations (*n* = 3). The result is a representative of two independent experiments. *p < 0.05 by ANOVA. **(C)** WT *E*. *japonica-*infected DH82 cells at 3 dpi were fixed and labeled with mouse anti-rRipE and goat anti-mouse IgG (AF488, green). DNA was stained with DAPI (pseudo-colored in red). N, nucleus. Scale bar, 10 µm. **(D, E)** Host cell-free WT *E*. *japonica* was fixed and double labeled with mouse anti-rRipE (AF488, green) and rabbit anti-P28 (AF555, red). The diameter of individual WT bacteria was measured using ImageJ software. **(D)** Size distributions of P28-labeled *E*. *japonica* with or without RipE labeling. RipE^+^, RipE-positive, and P28-positive (green) bacteria. RipE^−^, RipE-negative and P28-positive bacteria. Vertical bars indicate the mean and standard deviation. ****, Significantly different (*p* < 0.0001) by two-tailed Student’s *t*-test (total bacteria, *n* = 565). **(E)** The percentage of RipE^+^ bacteria in two size groups: >0.5 µm and <0.5 µm. Data indicate the mean ± standard deviation of immunofluorescence images (*n* = 6) from Panel **(D)** ****, Significantly different (*p* < 0.0001) by two-tailed Student’s *t*-test. **(F)** Double immunofluorescence labeling of WT *E*. *japonica-*infected DH82 cells with mouse anti-rRipE (AF488, green) and rabbit anti-*Ehrlichia* CtrA (AF555, red). Merge, fluorescence images merged with differential interference contrast (DIC) image. Scale bar, 50 µm. **(G)** Pearson’s correlation coefficient (*R* = 0.74) of RipE and CtrA in WT *E*. *japonica* morulae in DH82 cells from panel **(F)** as determined by using ImageJ software with the Coloc 2 plugin.

Western blotting with a mouse antiserum developed against full-length recombinant RipE (rRipE) demonstrated that native RipE was expressed by WT *E. japonica* but was indeed absent in the Δ*ripE* mutant (**Figure 2A**). However, in agreement with previous growth curve analysis by PCR based on *Ehrlichia* 16S rRNA (Bekebrede et al., 2020), the growth of WT and Δ*ripE E. japonica* in DH82 cells was also similar as determined by Western blotting with an antibody (Ohashi et al., 1998) against the recombinant *Ehrlichia* major outer-membrane protein P28 (rP28) (**Figure 2A**). Inside host cells, *Ehrlichia* spp. undergo a biphasic developmental cycle (Zhang et al., 2007; Rikihisa, 2010), i.e., a nonreplicating infectious dense-core (DC) cell form (0.4–0.6 µm) and a replicating noninfectious reticulate cell (RC) form (>0.8 µm) (Popov et al., 1995). To determine the expression of *ripE* mRNA by *E. japonica*, synchronized cultures of WT *E. japonica*-infected DH82 cells were established as previously described (Liu et al., 2012). The *E. japonica* growth curve was first determined based on reverse transcription-qPCR (RT-qPCR) analysis comparing the levels of *Ehrlichia* 16S rRNA with dog *GAPDH* (**Figure 2B**). The results revealed a lengthy lag phase of *Ehrlichia* growth up to 36 h pi, followed by slow growth between 36 and 48 h and exponential growth between 60 and 84 h pi (**Figure 2B**). RT-qPCR using *ripE*-specific primers (**Supplementary Table S1**) demonstrated that the expression of *ripE* mRNA peaked at 48–60 h pi in synchronous cultures prior to the exponential growth (**Figure 2B**). Immunofluorescence microscopy showed that, at 3 days pi, the majority of *E. japonica*-containing morulae were positive for RipE (**Figure 2C**). To further characterize RipE expression among individual *Ehrlichia* of various sizes, host cell-free *E. japonica* was purified and immunofluorescence staining was performed using antibodies against RipE and P28 or CtrA, a transcription factor of the two-component system of *Ehrlichia*, which is primarily expressed at the DC stage (Cheng et al., 2011). Quantitation of RipE-expressing *E. japonica* (RipE^+^) among all P28-positive *Ehrlichia* showed that the majority (~80%) were in DC form, with a diameter of <0.5 μm (**Figures 2D, E**). Similarly, there was a significant correlation between RipE- and CtrA-positive *Ehrlichia* morulae (**Figures 2F, G**).

### RipE is expressed by *E. japonica* in mice, and Δ*ripE* proliferates less in the blood and tissues than WT

**Figure 3:**
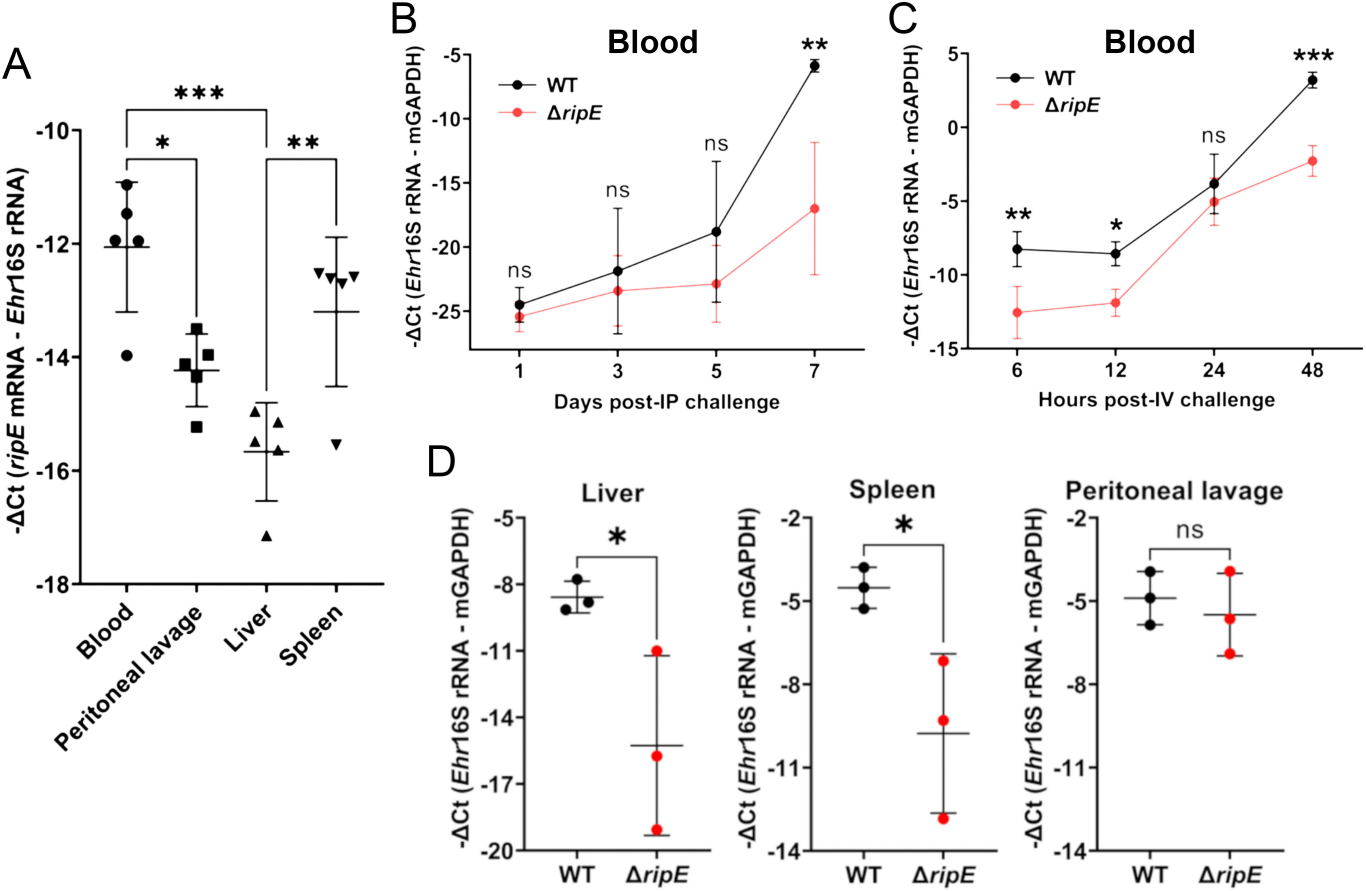
RipE is expressed by *E*. *japonica* in mice and the Δ*ripE* mutant has reduced infection in the blood and tissues compared to WT. **(A)** *ripE* mRNA expression of WT *E*. *japonica* in mouse tissues at 7 days pi. ICR mice were inoculated i.p. with infected DH82 cells containing ~24,000 WT *E*. *japonica*. Relative amount of *ripE* mRNA was analyzed by RT-qPCR and normalized against *Ehrlichia* (*Ehr*) 16S rRNA. Data indicate the mean ± standard deviation (*n* = 5). Results were analyzed with one-way ANOVA followed by Tukey’s multiple comparisons. ***p < 0.001, **p < 0.01, *p < 0.05. **(B)** Temporal bacteria loads (*Ehr* 16S rRNA) in the blood of C57BL/6 mice i.p. inoculated with infected DH82 cells containing ~1,000 WT or Δ*ripE E. japonica* by qPCR. **(C)** Temporal bacteria loads (*Ehr* 16S rRNA) in the blood of C57BL/6 mice i.v. inoculated with 1 × 10^7^ host cell-free WT or Δ*ripE E*. *japonica* by RT-qPCR. **(B, C)** Input DNA or RNA was normalized by mouse GAPDH. Data indicate the mean ± standard deviation (*n* = 3). Results were analyzed with repeated-measures ANOVA followed by Šídák multiple comparisons. ***p < 0.001, **p < 0.01, *p < 0.05, ns, not significantly different. **(D)** Bacteria loads in the tissues of C57BL/6 mice i.p. inoculated with infected DH82 cells containing ~100,000 WT or Δ*ripE E*. *japonica*. *Ehr* 16S rRNA was analyzed by RT-qPCR and normalized by mouse GAPDH mRNA. Data indicate the mean ± standard deviation (*n* = 3). *p < 0.05, ns, not significantly different by the Student’s *t*-test.

Upon intraperitoneal (i.p.) inoculation, *E. japonica* expressed *ripE* mRNA in various tissues of mice and levels were higher in the blood and spleen than in the liver and peritoneal lavage at day 7 pi (**Figure 3A**). To investigate the time course of proliferation of Δ*ripE* bacteria *in vivo*, mice were inoculated i.p. with Δ*ripE* or WT *E. japonica*, and bacterial load in blood samples was determined by qPCR during the early infection period. Up to day 5 pi, bacterial load did not differ significantly between the Δ*ripE*- and WT-infected mouse groups (**Figure 3B**). From days 5 to 7 pi, however, the bacterial load of WT *E. japonica* was significantly greater than that of Δ*ripE E. japonica* (**Figure 3B**). *E. japonica* infection in mouse blood via i.p. injection involves multiple steps, including the infection of resident macrophages in the peritoneal cavity and the subsequent spread to circulating blood. To examine *E. japonica* proliferation directly in the blood, mice were inoculated intravenously (i.v.) through the retro-orbital venous plexus with host cell-free Δ*ripE* or WT *E. japonica*. RT-qPCR data showed that, as early as 6 h pi, the abundance of WT *E. japonica* in the blood was significantly greater than that of Δ*ripE*, which remained higher throughout the 2-day infection time course (**Figure 3C**). In addition, mice inoculated i.v. with WT *E. japonica* showed signs of severe illness at day 3 pi and were moribund or had died by day 4, whereas mice inoculated with Δ*ripE* did not exhibit any signs of illness at day 4 pi.

To better understand the role of RipE during the early phase of infection, we inoculated mice (i.p.) with ~1,000-fold greater numbers of Δ*ripE* or WT *E. japonica* than used for the experiment shown in **Figure 2B**, followed by euthanasia and tissue sampling for subsequent RT-qPCR analysis. As early as 3 days pi, the bacterial load of the Δ*ripE* mutant was significantly lower in the liver and spleen compared with WT *E. japonica* (**Figure 3D**); for peritoneal lavage, however, the difference was not statistically significant (**Figure 3D**). Taken together, these data suggested that RipE may be involved in systemic infection and bacterial spread to various organs via blood.

### Extracellular Δ*ripE E. japonica* loses infectivity more rapidly and has lower ATP content than WT *E. japonica*

**Figure 4:**
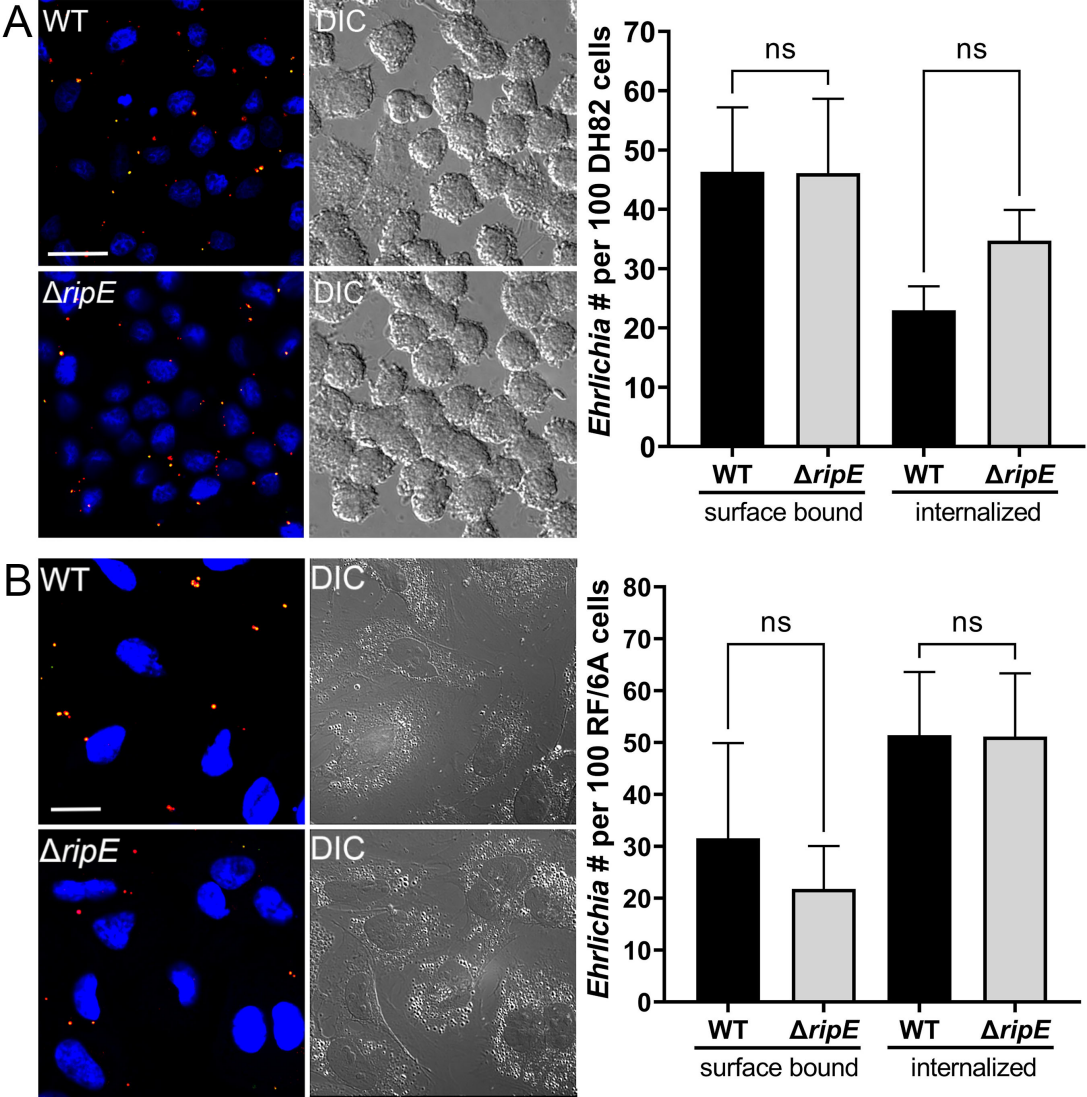
Host cell-free WT and Δ*ripE E*. *japonica* showed no difference in binding and entering mammalian cells in culture. (Left) Immunofluorescence image showing host cell-free WT and Δ*ripE E*. *japonica* incubated with DH82 for 30 min **(A)** or RF/6A cells for 60 min **(B)**. Cells were fixed and stained with anti-P28 (AF488, green), and then stained with anti-P28 (AF555, red) with saponin permeabilization. Scale bar, 20 µm. (Right) The numbers of bound bacteria (yellow) and internalized bacteria (red) in 100 cells were scored in ImageJ software. ns, not significantly different by the Student’s *t*-test (*n* = 3).

As an obligatory intracellular bacterium, *E. japonica* must bind and enter host cells (Rikihisa, 2010). We therefore examined whether RipE plays a role in this process. Using two-step fluorescent labeling, we quantified host cell surface-bound (non-internalized) vs. internalized *Ehrlichia* upon incubation with DH82 and RF/6A cells as previously described (Mohan Kumar et al., 2013), revealing no significant difference in bacterial binding or entry between Δ*ripE* and WT *E. japonica* (**Figure 4**).

**Figure 5:**
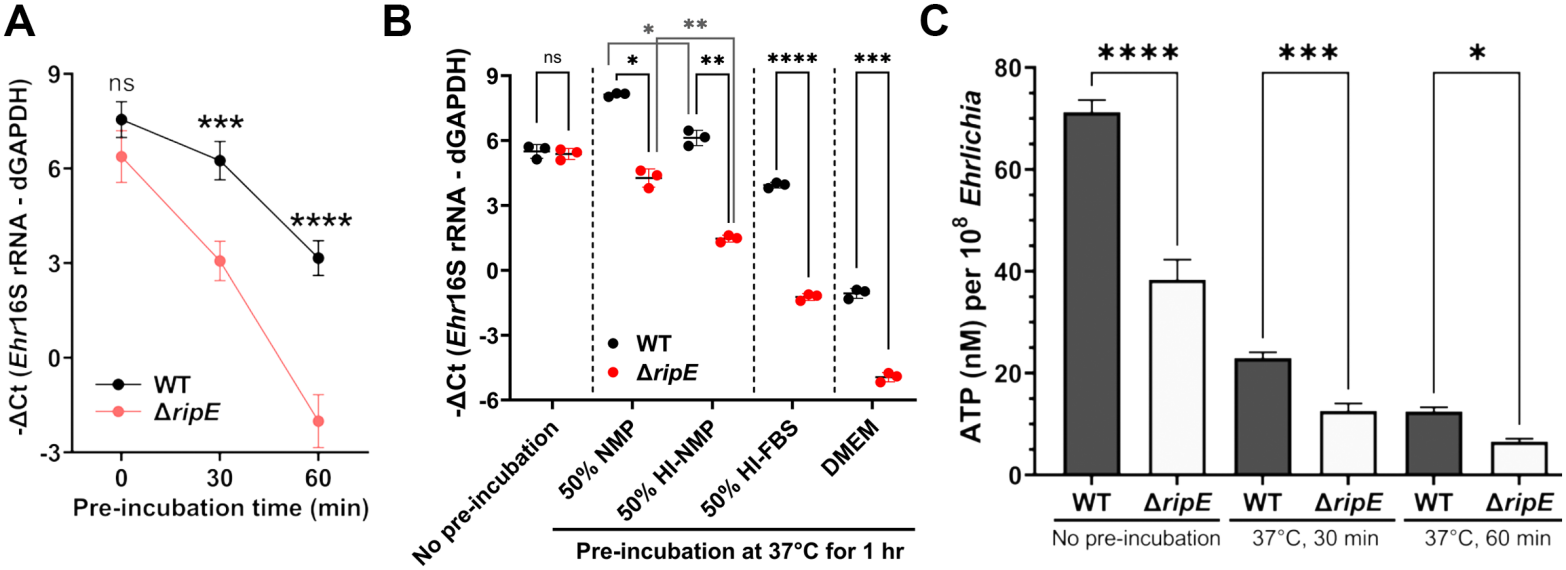
Host cell-free Δ*ripE E*. *japonica* more rapidly loses infectivity and ATP reserve in culture than WT *E*. *japonica.* **(A)** Temporal loss of infectivity of host cell-free WT or Δ*ripE E*. *japonica* preincubated at 37°C in culture medium (DMEM with 4% heat-inactivated FBS) in DH82 cells (MOI 2,500–3,000). **(B)** Infectivity of host cell-free WT and Δ*ripE E. japonica* preincubated at 37°C for 1 h in different media in DH82 cells (MOI 500–1,000). NMP, normal mouse serum; HI, heat inactivated at 56°C for 30 min. **(A, B)** The infected DH82 cells were harvested at 2 dpi for *Ehrlichia* 16S rRNA-specific RT-qPCR. Input RNA was normalized by dog GAPDH (dGAPDH). Data indicate the mean ± standard deviation (*n* = 3). Results were analyzed with repeated-measures ANOVA followed by Šídák multiple comparisons **(A)** or one-way ANOVA followed by Tukey’s multiple comparisons **(B)**. ****p < 0.0001, ***p < 0.001, **p < 0.01, *p < 0.05, ns, not significant different. **(C)** Levels of ATP in host cell-free WT or Δ*ripE E*. *japonica* incubated in 4% heat-inactivated FBS for 0, 30, and 60 min. ATP levels were determined using the Luminescent ATP Detection Assay Kit normalized with the number of *Ehrlichia* determined by *Ehr*16S rRNA gene-specific qPCR. Data indicate the mean ± standard deviation of triplicate samples. ****p < 0.0001, ***p < 0.001, *p < 0.05, significantly different based on one-way ANOVA followed by Tukey’s multiple comparisons.

*Ehrlichia* must be released from infected host monocytes/macrophages and survive the extracellular stage to infect new host cells and spread to other tissues/organs. Therefore, we next compared the infectivity of host cell-free *Ehrlichia* that had been briefly preincubated in culture medium up to 60 min. Without preincubation, freshly isolated Δ*ripE* and WT *E. japonica* had the same infectivity on DH82 cells (**Figures 5A, B**). Both Δ*ripE* and WT *E. japonica* progressively lost their infectivity in the host cell-free medium, but Δ*ripE* lost its infectivity significantly faster than WT did (**Figure 5A**).

Extracellular *Ehrlichia* is exposed to the serum, and serum complement resistance has been reported for certain *Rickettsia* spp (Riley et al., 2018). Thus, to determine the effects of serum on extracellular *Ehrlichia*, and whether *E. japonica* is complement-resistant, we preincubated host cell-free *Ehrlichia* (WT or Δ*ripE*) in 50% normal mouse plasma (NMP), which mimics mouse blood, and 50% heat-inactivated NMP; negative controls include 50% heat-inactivated fetal bovine serum (FBS, used in cell culture of *E. japonica*) and serum-free Dulbecco’s modified Eagle medium (DMEM). The presence of serum in the culture media profoundly protected the infectivity of extracellular *Ehrlichia* regardless of the type of serum in both WT and Δ*ripE* mutant (**Figure 5B**). The infectivity of *E. japonica* was significantly lower by heat inactivation of serum components (**Figure 5B**), suggesting that *E. japonica* is complement-resistant, and heat-labile plasma components are rather beneficial for extracellular *Ehrlichia*. The Δ*ripE* mutant lost its infectivity significantly more than WT *E. japonica* in each medium tested, including in the absence of serum (**Figure 5B**), implying that the presence of RipE helps maintain the infectivity of extracellular *Ehrlichia* via a mechanism that is independent of factors in the serum.

Freshly isolated *Ehrlichia* can transiently produce the essential chemical-energy component adenosine triphosphate (ATP) (Weiss et al., 1989). We therefore quantified the time course of the total ATP in host cell-free *Ehrlichia* using the Luminescent ATP Detection Assay Kit (see Materials and Methods). The total ATP levels in host cell-free *Ehrlichia* were significantly higher in WT than in the Δ*ripE* mutant regardless of the incubation time in the culture medium containing 4% heat-inactivated FBS (**Figure 5C**).

### Genomic complementation of the Δ*ripE* mutant partially restores the *Ehrlichia* ATP level, and overexpression of *ripE* increases the ATP level and *in vivo* virulence

**Figure 6:**
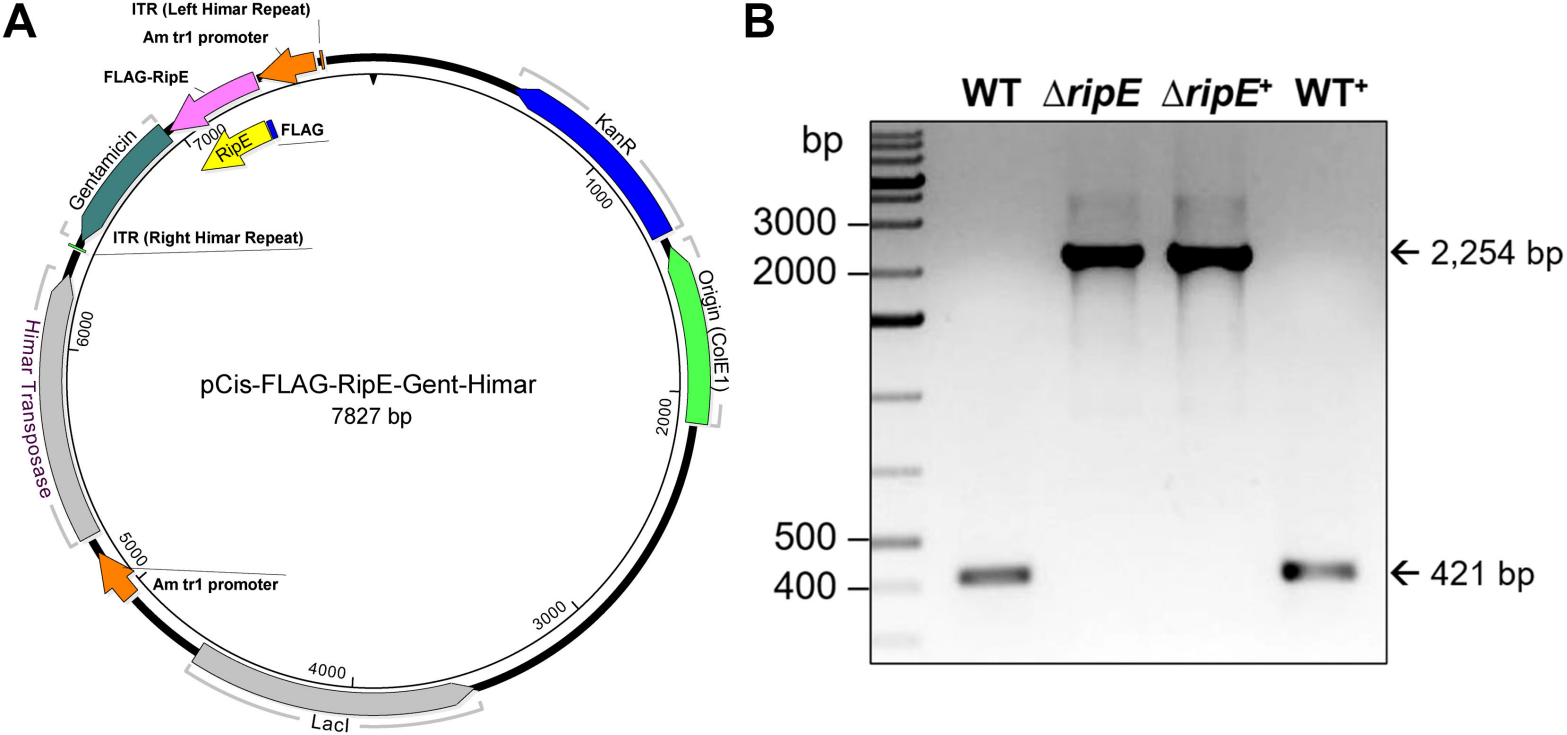
Plasmid construction for genomic complementation of *ripE* in Δ*ripE E. japonica* and overexpression of *ripE* in WT *E*. *japonica*. **(A)** pCis-FLAG-RipE-Gent-Himar plasmid map. **(B)** *ripE* gene-specific flank PCR for Δ*ripE* and Δ*ripE* ^+^ clonality verification. Corresponding amplicon sizes for WT (421 bp) and the original Himar1 insertion disrupted *ripE* gene (2,254 bp) are indicated by arrows.

To test if RipE produced within *Ehrlichia* counteracts reduced ATP levels in the extracellular Δ*ripE* mutant and increases *Ehrlichia* virulence in mice, we performed genomic complementation of the Δ*ripE* mutant with *ripE* or overexpression of *ripE* in WT *E. japonica* bacteria by Himar1 mutagenesis with the newly constructed pCis-FLAG-RipE-Gent-Himar plasmid, which encodes a gentamicin resistance gene and FLAG-RipE (**Figure 6A**). A total of three transformed WT (WT^+^; genomic insertion of Himar 1, including Eja^+^_0814_, Eja^+^_1020_, and Eja^+^_1231_) and one transformed Δ*ripE* (Δ*ripE* ^+^; genomic insertion of Himar1, H59^+^_1014_) *E. japonica* were obtained and stably cultured in DH82 cells (Bekebrede et al., 2020) (**Table 2**). In subsequent experiments, WT^+^ (Eja^+^_0814_) was used as a representative of *E. japonica* overexpressing RipE, whereas Δ*ripE* ^+^ (H59^+^_1014_) was used to represent rescued Δ*ripE E. japonica*. *ripE*-gene-specific flanking PCR confirmed the original Himar1 insertion in Δ*ripE* and Δ*ripE* ^+^, whereas the *ripE* gene was intact in WT and WT^+^ (**Figure 6B**).

**Table 2:** Mutants of *E. japonica* transformed with pCis-FLAG-EHF0962-Gent-Himar A7 plasmid^1^.

Mutant ID	Genomic insertion site	Insertion orientation^2^	Insertion in ORF/ORF length (bp)	Disrupted gene locus^1^	Disrupted gene product
**Eja^+^_0814_** (WT^+^)	16,259	−	**99**/810	EHF_0017 (16358…15549)	Pyrroline-5-carboxylate reductase dimerization domain-containing protein (GenBank accession no. WP_044193877.1)
**H59^+^_1014_** (Δ*ripE* ^+^)	821,340	−	**1,317**/2,415	EHF_0723 (822657…820243)	DNA mismatch repair protein MutS (GenBank accession no. WP_044195176.1)
**Eja^+^_1020_**	150,896	−	**1,536**/2,178	EHF_0140 (149360…151537)	Hypothetical protein (GenBank accession no. WP_232228951.1)
**Eja^+^_1231_**	607,646	+	**409**/600	EHF_0545 (607237 …607836)	DUF3023 domain-containing protein (GenBank accession no. WP_232228923.1)

^1^GenBank accession number of *E. japonica* HF genome: NZ_CP007474.1;

ORF, open reading frame; *Eja*, *Ehrlichia japonica*.

^2^The + or − indicates the insertion in forward or reverse orientation of the disrupted ORF, respectively.

**Figure 7:**
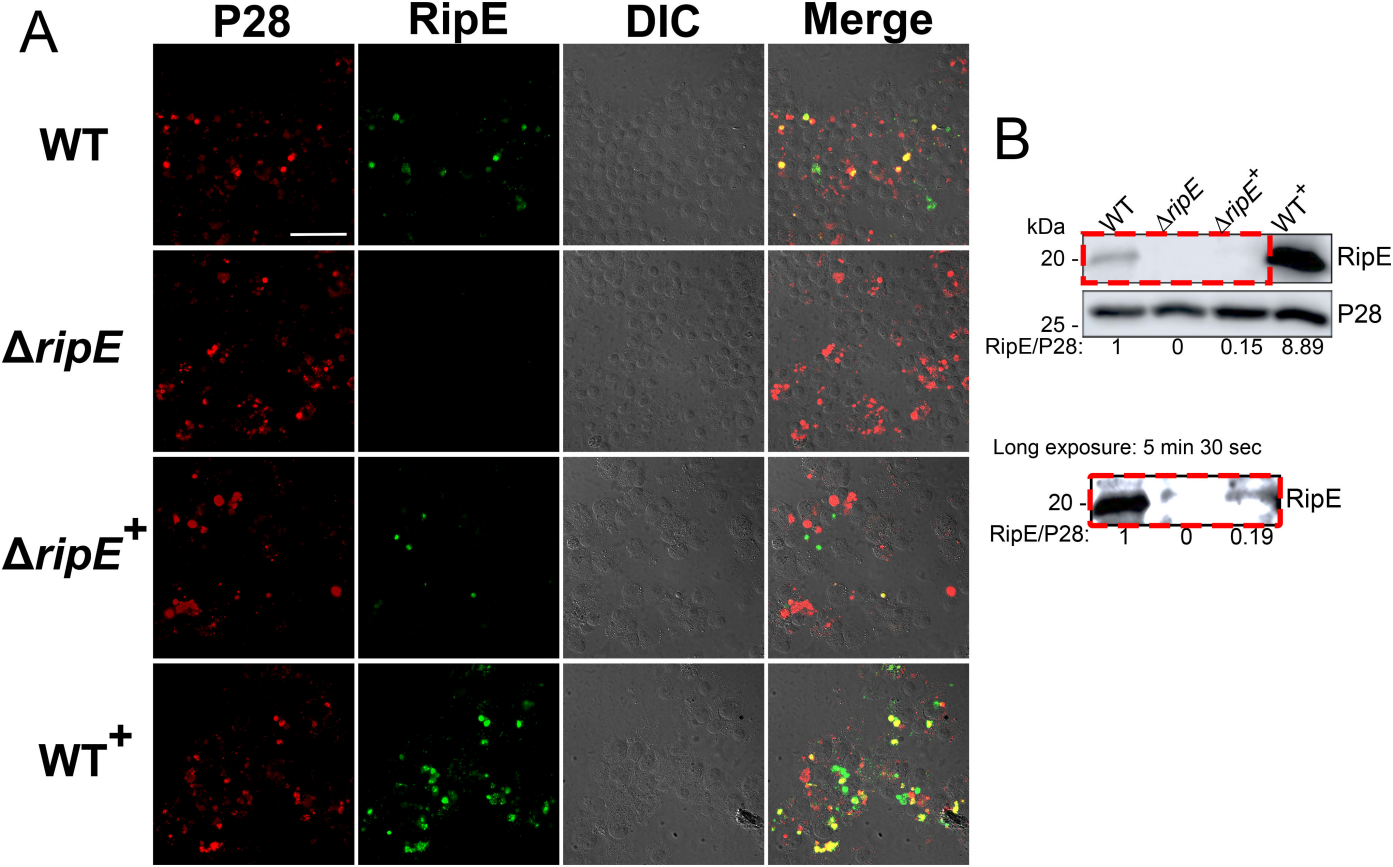
Genomic complementation or overexpression of *ripE.* **(A)** Double immunofluorescence labeling of WT, Δ*ripE*, RipE-complemented Δ*ripE* (Δ*ripE* ^+^), and RipE-overexpressing WT (WT^+^) *E*. *japonica*-infected DH82 cells with rabbit anti-*Ehrlichia* P28 (AF555, red) and mouse anti-rRipE (AF488, green). Merge, fluorescence image merged with differential interference contrast (DIC) image. Scale bar, 50 µm. **(B)** Lysates of WT, Δ*ripE*, Δ*ripE* ^+^, and WT^+^ *E*. *japonica*-infected DH82 cells were analyzed by Western blotting using anti-rRipE and anti-P28. Numbers below each band are relative ratios of RipE band intensities normalized by P28, with the ratio of WT set as 1. Red dashed box on the lower panel showed the image under long exposure time (5 min and 30 s).

Immunofluorescence staining revealed that WT^+^ *E. japonica* overexpressed RipE within more than 80% of the *Ehrlichia* morulae, much higher than what was observed for WT *E. japonica*, which typically contained 20-30% RipE-positive morulae (**Figure 7A**). Genomic complementation of the Δ*ripE* mutant partially restored *ripE* expression in Δ*ripE* ^+^ *E. japonica*, although RipE abundance was relatively low, i.e., less than 5% RipE-positive morulae (**Figure 7A**). Western blotting confirmed that WT^+^ *E. japonica* overexpressed approximately 9-fold or 60-fold more RipE protein than WT or Δ*ripE* ^+^ *E. japonica* in culture, respectively (**Figure 7B**).

**Figure 8:**
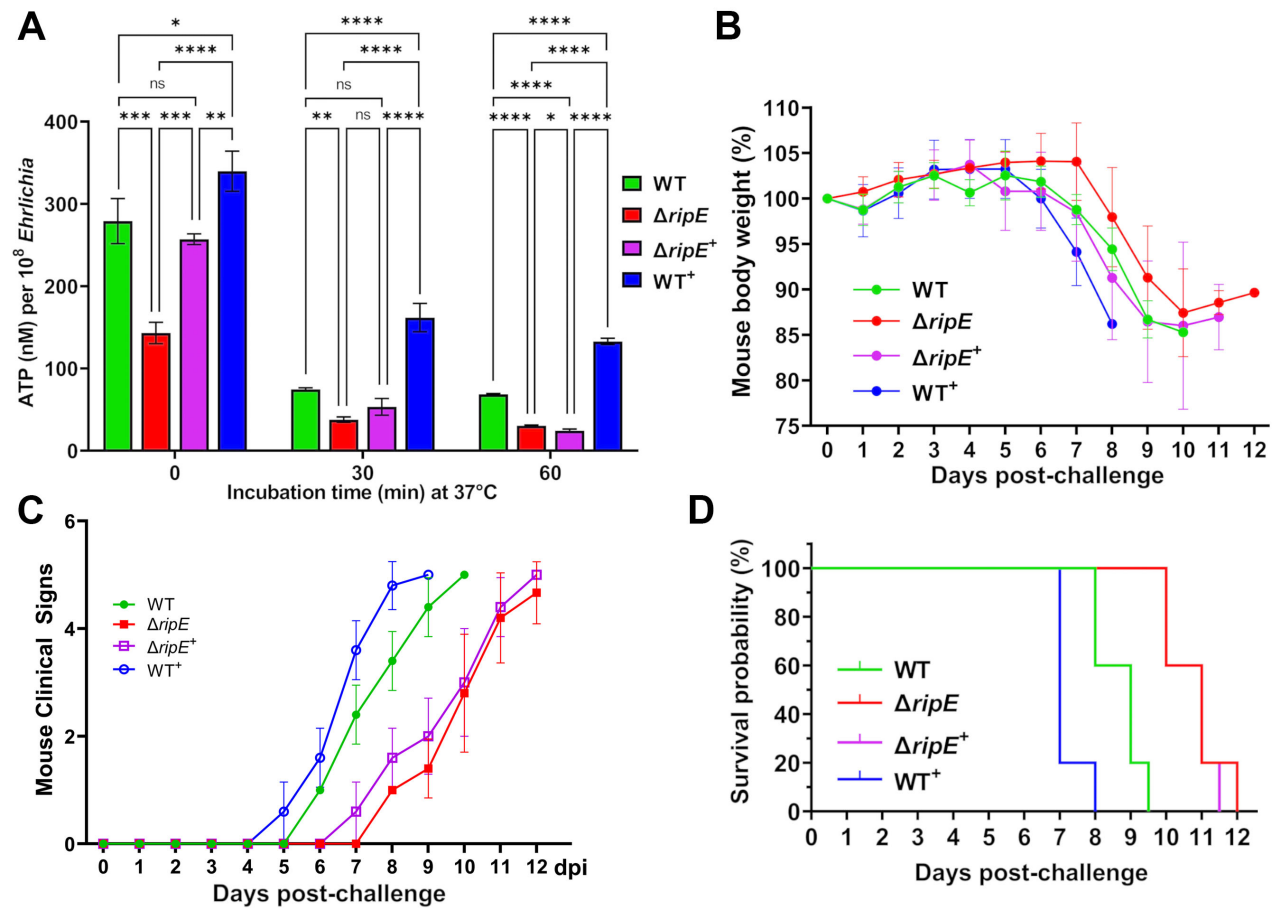
RipE expression increases *Ehrlichia* ATP reserve and *in vivo* virulence. **(A)** Levels of ATP in host cell-free WT, Δ*ripE*, Δ*ripE^+^*, and WT^+^ *E*. *japonica* incubated in 4% heat-inactivated FBS for 0, 30, and 60 min. ATP levels were determined using the Luminescent ATP Detection Assay Kit and normalized with bacterial numbers determined by *Ehrlichia* 16S rRNA gene-specific qPCR. Data indicate mean ± standard deviation of triplicate samples. ****p < 0.0001; ***p < 0.001; **p < 0.01; *p < 0.05; significantly different, ns, not significantly different based on one-way ANOVA followed by Tukey’s multiple comparisons. **(B–D)** ICR mice (4-week-old male, five mice per group) were i.p. inoculated with WT, Δ*ripE*, Δ*ripE* ^+^, and WT^+^ *E*. *japonica-*infected DH82 cells. The number of *Ehrlichia* inoculated was determined by *Ehrlichia* 16S rRNA-specific qPCR: WT, 5,195 bacteria/mouse; Δ*ripE*, 5,902; Δ*ripE* ^+^, 4,654; and WT^+^, 9,490. **(B)** Body weight changes. **(C)** Signs of illness (ruffled fur coat, hunched back, and squinty eyes, and reluctance to move) were assigned the rating of 0 to 4 for severity and 5 for death. Statistical analysis was performed using two-way repeated-measures ANOVA and showed that clinical signs of the infected mice were significantly more severe in those challenged with *E*. *japonica* expressing RipE in the order of WT^+^ > WT >> Δ*RipE* ^+^ than the Δ*RipE* mutant that does not express RipE (*p* < 0.01). Data indicate mean ± standard deviation of five mice. **(D)** Kaplan–Meier survival curves. Compared with mice inoculated with WT, mice inoculated with Δ*ripE* and Δ*ripE* ^+^ survived significantly longer (*p* < 0.01), whereas mice inoculated with WT^+^ died significantly earlier (*p* < 0.01) analyzed by log-rank (Mantel–Cox) test (*n* = 5). There was no significant difference between Δ*ripE* and Δ*ripE* ^+^ (*p* = 0.663).

To test if RipE amounts within *Ehrlichia* correlate with ATP levels in extracellular *Ehrlichia*, the time course of ATP loss in extracellular WT, Δ*ripE*, and the transformed *E. japonica* in the culture medium that contains 4% heat-inactivated FBS was examined. Without incubation at 37°C, WT^+^ *E. japonica* had the highest average ATP level, followed by WT, Δ*ripE* ^+^, and Δ*ripE E. japonica* (**Figure 8A**). After incubation at 37°C for 30 or 60 min, however, the ATP levels dropped greatly in all groups, although WT^+^ *E. japonica* maintained a significantly higher ATP level compared to WT, Δ*ripE* ^+^, and Δ*ripE E. japonica* (**Figure 8A**).

To test whether intrinsic RipE levels correlate with *Ehrlichia* virulence *in vivo*, ICR mice were inoculated i.p. with WT, Δ*ripE*, Δ*ripE* ^+^, or WT^+^ *E. japonica* (five mice per group). Mice were weighed daily and monitored for clinical signs. Although the body weight changes were not dramatically different among the groups (**Figure 8B**), the onset and severity of clinical signs of the infected mice were much earlier and significantly more severe in those challenged with *E. japonica* expressing RipE in the order of WT^+^ > WT >> Δ*ripE* ^+^ than the Δ*ripE* mutant that does not express RipE (**Figure 8C**). Kaplan–Meier survival curves showed that WT^+^ *E. japonica* was significantly more virulent than WT and Δ*ripE E. japonica* (**Figure 8D**); however, loss of virulence in Δ*ripE E. japonica* was not restored in Δ*ripE* ^+^ *E. japonica* (**Figure 8D**), in agreement with the observed low level of RipE expression (**Figures 7A, B**).

### RipE is a bacterial outer-membrane protein

**Figure 9:**
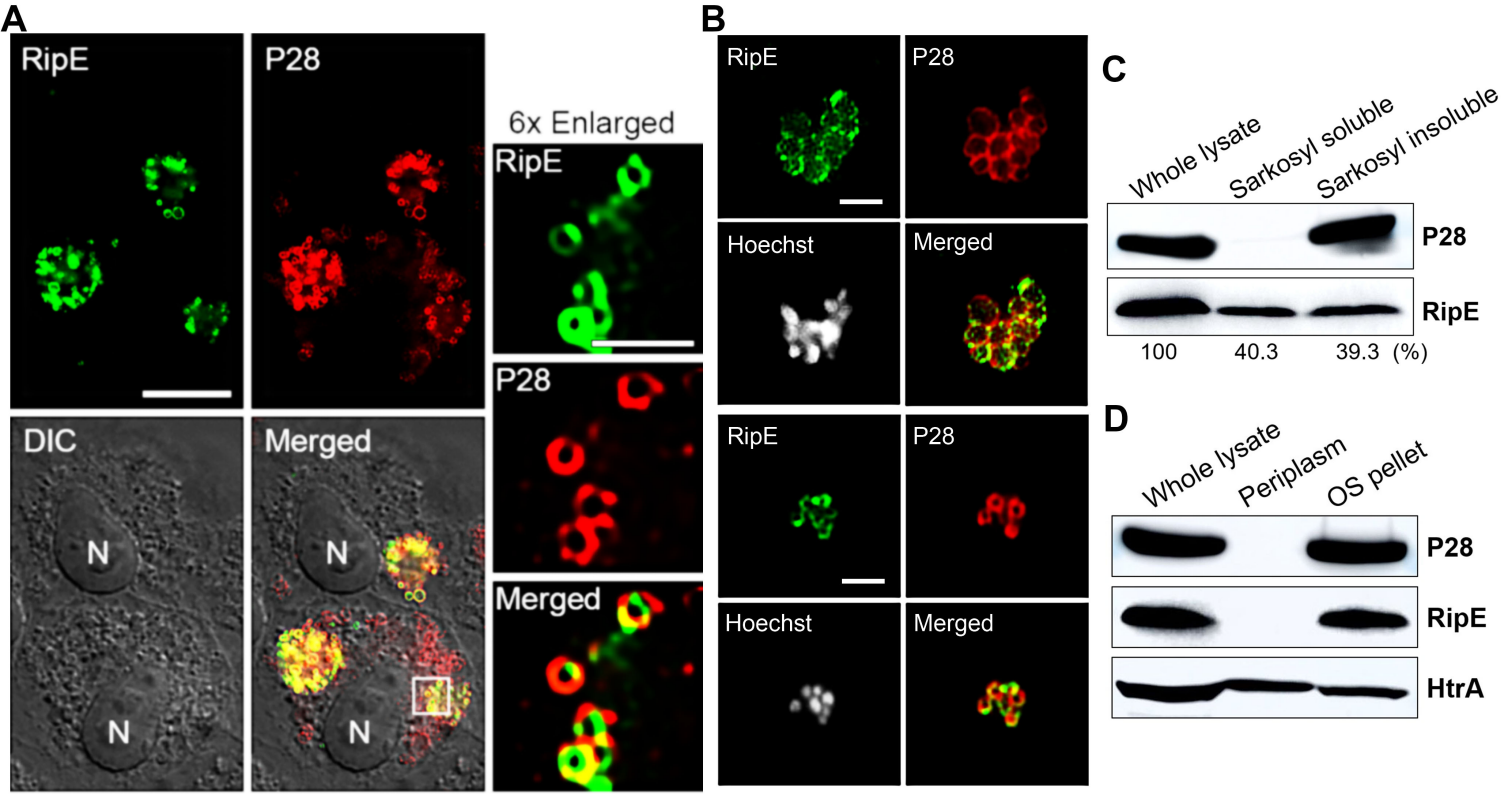
RipE is present on the outer membrane of *Ehrlichia*. **(A)** Immunofluorescence labeling of *E*. *japonica* overexpressing RipE (WT^+^) in DH82 cells with mouse anti-rRipE (AF488, green) and rabbit anti-P28 IgG (AF555, red) with membrane permeabilization by saponin. Scale bar, 10 µm. Merged, fluorescence images merged with DIC image. N, nucleus. Boxed area was enlarged 6× on the right. Scale bar, 2 µm. **(B)** Immunofluorescence labeling of spontaneously released WT *E*. *japonica* with mouse anti-rRipE (AF488, green) and rabbit anti-P28 IgG (AF555, red) without membrane permeabilization. DNA was stained by Hoechst 33342 and pseudocolored gray. Scale bar, 2 µm. **(C)** Western blot analysis of the whole lysate, and Sarkosyl-soluble and -insoluble (outer membrane) fractions of purified WT *Ehrlichia* with mouse anti-rRipE and rabbit anti-P28. Numbers below each band are percentages of RipE protein relative to the whole lysate (set as 100%). **(D)** Western blot analysis of the whole lysate, soluble (periplasm), and insoluble fractions by osmotic shock (OS) of purified WT *E*. *japonica* with mouse anti-rRipE and rabbit anti-*Ehrlichia* P28 and anti-HtrA sera.

To study mechanisms by which RipE is involved in maintaining the infectivity and ATP levels in extracellular *E. japonica*, we analyzed the subcellular localization of RipE in *E. japonica* using DeepLocPro, an AI-based prokaryotic deep-learning algorithm (Izadi-Pruneyre et al., 1999; Moreno et al., 2024). By this analysis, RipE protein was predicted to localize to the bacterial membranes or be secreted. Immunofluorescence labeling using RipE- and outer-membrane protein P28-specific antibodies demonstrated that RipE colocalized with P28, showing ring-like labeling patterns in both *E. japonica*-infected DH82 cells that had been permeabilized with saponin (**Figure 9A**) and in host cell-free WT *E. japonica* without permeabilization (**Figure 9B**). These results suggested that RipE was expressed on the *Ehrlichia* surface.

To confirm the surface localization of RipE, *E. japonica* was fractionated by the sodium dodecyl sarcosine (Sarkosyl) solubilization method, which is an amphiphilic, ionic detergent that has been used to isolate the *E. chaffeensis* outer-membrane fraction (Ohashi et al., 1998). Sarkosyl fractionation of purified WT *E. japonica* resulted in soluble (cytosol, inner membrane, and periplasm) and insoluble (outer membrane) fractions. Using *Ehrlichia* P28 as a control, the results showed that native RipE of WT *E. japonica* was present in both Sarkosyl-soluble and Sarkosyl-insoluble fractions at a ratio of approximately 1:1 (**Figure 9C**), confirming that ~50% of RipE localizes to the outer membrane. To test if the remaining 50% of RipE is present in the periplasm of *E. japonica*, the osmotic shock method (Cheon et al., 2021) was adapted for isolating *Ehrlichia* periplasmic proteins (Kumagai et al., 2010). The results showed that, unlike the control *E. japonica* HtrA protein, which is a serine protease normally present in the *E. chaffeensis* periplasm and outer surface (Kumagai et al., 2010), RipE was absent in the periplasmic fraction (**Figure 9D**), suggesting that ~50% RipE is localized in the inner membrane or cytoplasm of *E. japonica*.

### rRipE induces a robust humoral response with the production of an *E. japonica-*neutralizing antibody and is a vaccine candidate for ehrlichiosis

**Figure 10:**
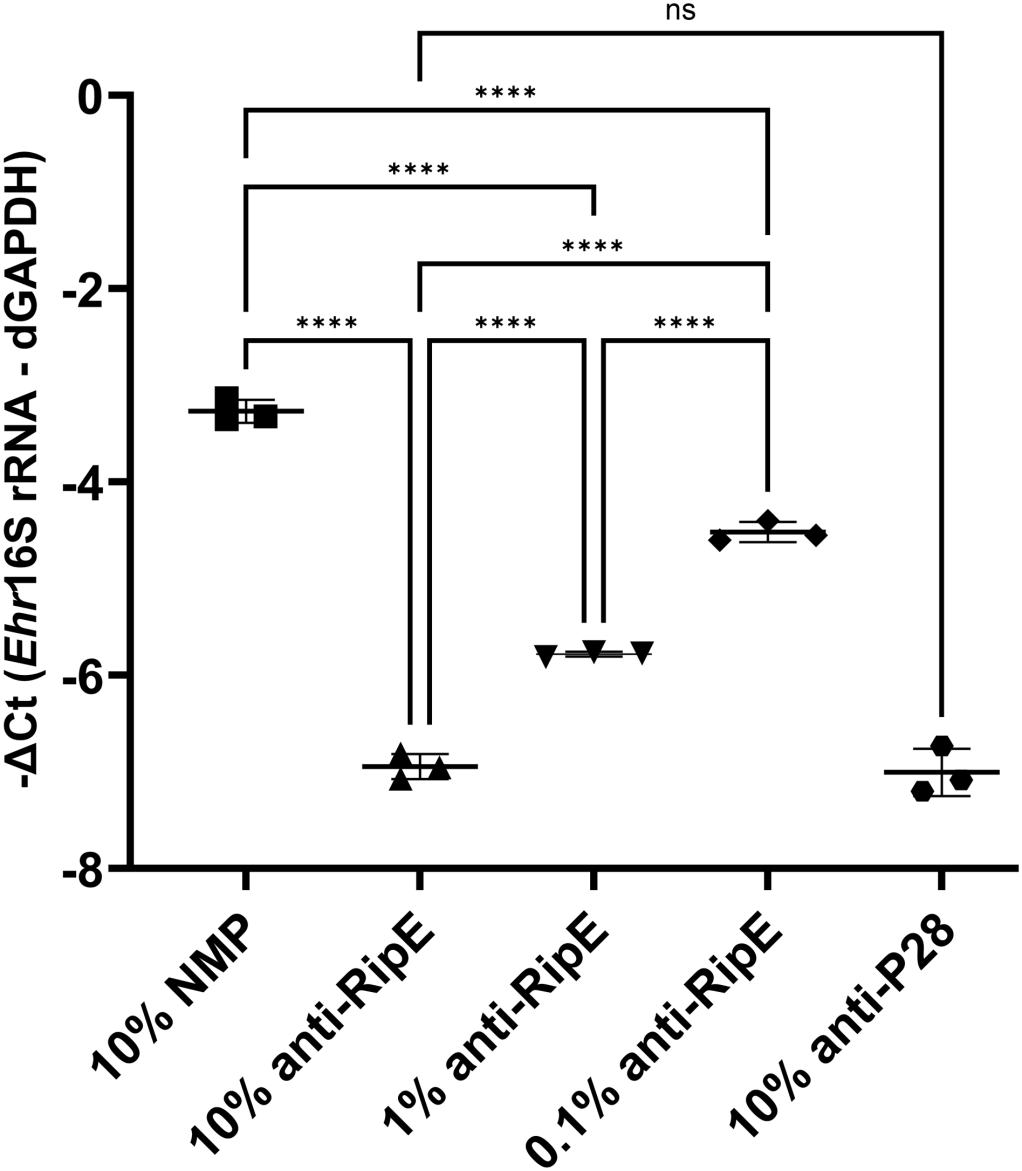
*In vitro* neutralization of *E. japonica* by anti-rRipE. Freshly isolated host cell-free WT *E. japonica* (~1,000 MOI) was preincubated with 10% normal mouse plasma (NMP), 10%, 1%, or 0.1% mouse anti-rRipE serum, or 10% rabbit anti-P28 serum at 37°C for 30 min. Sera and plasma were all diluted in the culture medium. The mixtures of *Ehrlichia* and antisera were added to DH82 cells and incubated at 37°C, and infection was determined at 48 hpi by *Ehrlichia* 16S rRNA gene-specific qPCR. Input DNA was normalized by dog GAPDH (dGAPDH). ****, significantly different (*p* < 0.0001); ns, not significantly different based on one-way ANOVA followed by Tukey’s multiple comparisons (*n* = 3). The result is representative of two independent experiments.

Anti-RipE antibody produced using rRipE recognized denatured protein by Western blotting (**Figures 2A**, **7B**, **9C, D**) and native protein by immunofluorescence staining (**Figures 2C, F**, **7A**, **9A, B**), indicating that rRipE is antigenic and capable of inducing a robust humoral response in mice. Indeed, *in vitro* neutralization experiments revealed that mouse anti-rRipE antisera could significantly reduce *E. japonica* infection *in vitro* in a dose-dependent manner compared to NMP (**Figure 10**). The neutralizing effect of the mouse anti-RipE antiserum for reducing *E. japonica* infection *in vitro* was similar to that of anti-rP28 (**Figure 10**), which can neutralize *E. chaffeensis* infection in immunocompetent mice (Ohashi et al., 1998) and protect severe combined immunodeficiency mice from fatal *E. chaffeensis* infection (Li et al., 2001).

**Figure 11:**
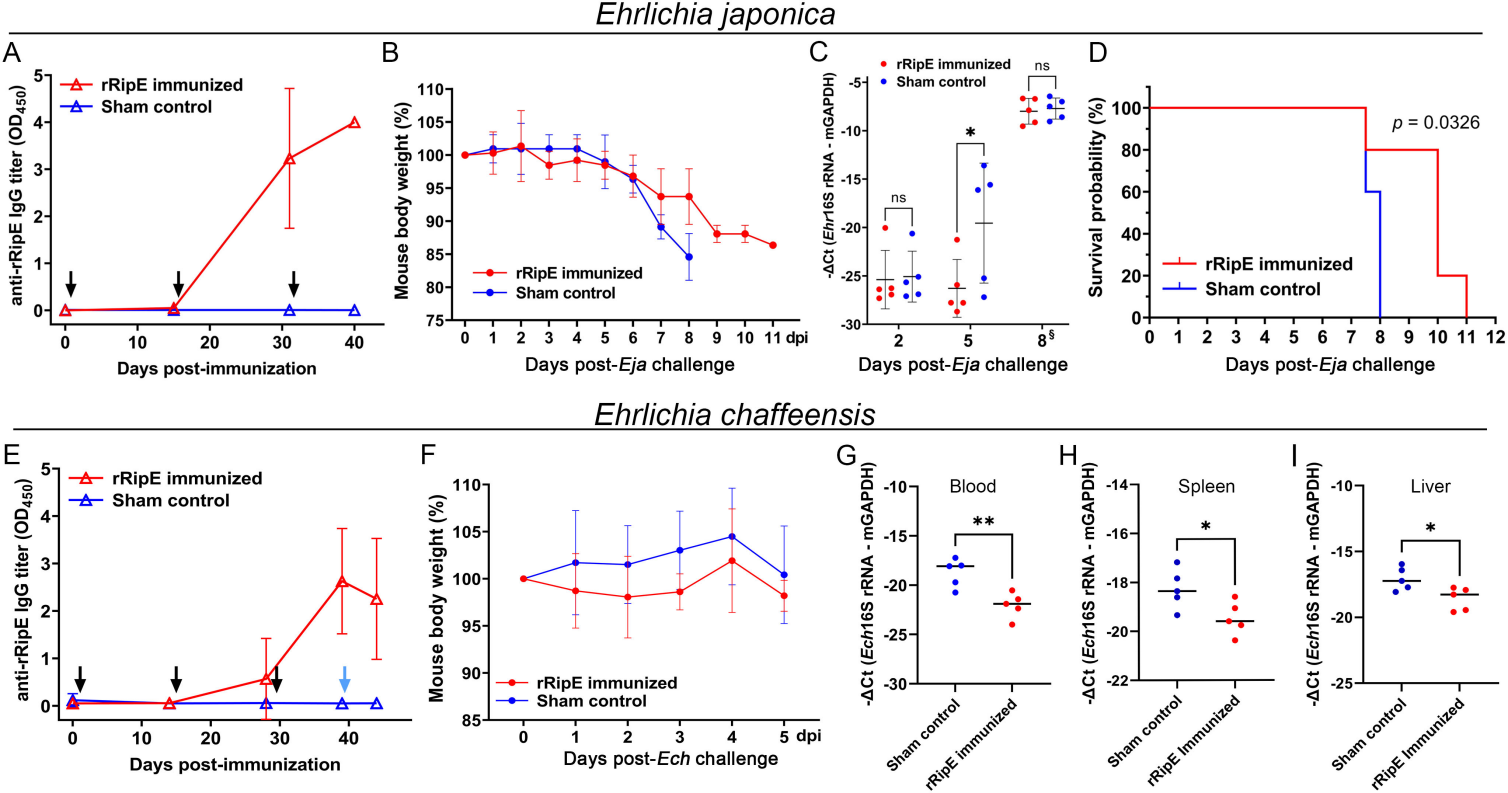
rRipE immunization increased the survival time of mice challenged with a lethal dose of *E*. *japonica*. ICR **(A–D)** or C57BL/6 **(E–I)** mice were subcutaneously injected with rRipE plus QuilA adjuvant (rRipE immunized) or QuilA alone (Sham control), and challenged by i.p. inoculation of infected DH82 cells containing a lethal dose of WT *E*. *japonica* (11,750) at 9 days post third immunization [*Eja*, **(A–D)**], or infected THP-1 cells containing *E*. *chaffeensis* (~120,000) at 11 days post third immunization [*Ech*, **(E–I)**], respectively. **(A, E)** Titers of rRipE-specific antibody in mice sera were measured by ELISA using rRipE as the antigen. Black arrows indicate days on which mice were vaccinated, and blue arrow denotes the day on which mice were challenged with *E*. *chaffeensis*. Data indicate the mean ± standard deviation (*n* = 5). **(B, F)** Mouse body weight changes in rRipE-immunized and sham-immunized mice upon challenge with WT *E*. *japonica* **(B)** or *E*. *chaffeensis* **(F)** (*n* = 5). **(C)** Bacterial loads of *E*. *japonica* in the blood of rRipE-immunized and control mice by *Ehrlichia* 16S rRNA gene-specific qPCR normalized by mouse GAPDH (mGAPDH). Data indicate the mean ± standard deviation (*n* = 5). Results were analyzed with repeated-measures ANOVA followed by Šídák multiple comparisons. *p < 0.05; **p < 0.01; ns, not significantly different. **^§^** Mouse blood samples were collected at 8 days pi or at the time of death (two control mice and one rRipE immunized mice were found dead a few hours before 8 days pi). **(D)** Kaplan–Meier survival curves for rRipE- and sham-immunized mice following lethal challenge with WT *E*. *japonica*. Compared with control mice, rRipE-immunized mice survived significantly longer (*p* < 0.05) by log-rank (Mantel–Cox) test (*n* = 5). **(G–I)** Bacterial loads of *E*. *chaffeensis* in the rRipE-immunized and sham-immunized mice on day 5 post-challenge were determined using *Ehrlichia* 16S rRNA gene normalized by mouse GAPDH (mGAPDH). **(G)** Blood samples, qPCR. **(H)** Spleen and **(I)** liver samples, RT-qPCR. The scatter plot shows the normalized *Ehrlichia* levels in individual mice, with the horizontal bar representing the mean value. **p < 0.01; *p < 0.05 by Student’s *t*-test.

To test whether the rRipE-induced *Ehrlichia-*neutralizing antibody could prevent fatal ehrlichiosis *in vivo*, we immunized five mice three times with rRipE at 2-week intervals followed by a lethal challenge with WT *E. japonica* through i.p. injection. An enzyme-linked immunosorbent assay (ELISA) demonstrated that all rRipE-immunized mice generated a significant rRipE-specific IgG after the third immunization (**Figure 11A**), whereas the sham-immunized controls did not. At day 7 post-WT *E. japonica* challenge, all sham control mice had lost more than 10% of body weight (**Figure 11B**) and appeared severely ill or moribund, characterized by ruffled haircoat, hunched back, squinty eyes, and reluctance to move, whereas rRipE-immunized mice experienced lesser body weight loss, with mice being relatively normal, alert, and responsive. Bacterial load in the blood of rRipE-immunized mice was significantly lower than in sham controls at 5 days pi (**Figure 11C**). At 7–8 days pi, however, bacterial load did not differ between the two groups (**Figure 11C**). Kaplan–Meier curves showed that rRipE-immunized mice survived significantly longer than the sham controls, although all mice eventually succumbed to fatal ehrlichiosis up to 11 days pi (**Figure 11D**).

As *E. japonica* and *E. chaffeensis* RipE share 46% amino acid identities (**Table 1**), we tested whether mice vaccinated with *E. japonica* rRipE could protect infection by the human ehrlichiosis agent *E. chaffeensis*. Mice immunization with *E. japonica* rRipE or sham control as described above was challenged i.p. with *E. chaffeensis*-infected DH82 cells (~1.2 × 10^5^ bacteria per mouse). Similarly, all rRipE-immunized mice generated an abundance of rRipE-specific IgG after the third immunization (**Figure 11E**). Mouse infection with *E. chaffeensis* did not exhibit any apparent clinical signs (weight loss, lethargy, anorexia, squinting eyes, or ruffled fur) throughout the experimental course (**Figure 11F**). However, *E. chaffeensis* loads in the blood, spleen, and liver samples of rRipE-immunized mice were significantly lower than those of sham controls at 5 days post-challenge (**Figures 11G–I**), suggesting that RipE can be a potential vaccine candidate for human ehrlichiosis.

## Discussion

As obligate intracellular bacteria, *Ehrlichia* spp. need to be extracellular in order to spread within the host and transmit between the susceptible cells. However, this process is poorly understood. The *Ehrlichia in vivo* virulence factor RipE (Bekebrede et al., 2020) is the first example of a bacterial molecule that helps maintain infectivity in the extracellular stage of *Ehrlichia* and its ATP levels (Bekebrede et al., 2020). RipE protein was expressed mostly at the DC stage of *E. japonica* and partially on the bacterial surface. There are clear correlations between RipE expression levels, bacterial ATP levels, and mouse virulence among WT *E. japonica* and Δ*ripE* mutants. For obligate intracellular bacteria, the dogma has been that the extracellular, resistant forms, such as the elementary bodies (EBs) of *Chlamydia trachomatis*, are metabolically dormant so that they can reserve relatively higher energy than reticular bodies (RBs) until they reach the next susceptible host cell (Tipples and McClarty, 1993). However, Omsland et al. (2012) showed that both EBs and RBs of *C. trachomatis* can perform *de novo* protein synthesis and generate ATP in axenic culture, and proteomics analysis suggests that EBs of *C. trachomatis* are metabolically active (Skipp et al., 2016), which provides them the advantage and the ability to rapidly deploy bacterial effectors immediately upon contact with host cell plasma membrane and establish infection (Cossé et al., 2018). Protein and DNA synthesis was also demonstrated in host cell-free *E. chaffeensis* organisms incubated in an axenic medium containing amino acids, nucleotides, and different energy sources, albeit the biosynthetic activity was mostly observed in RCs rather than in DCs (Eedunuri et al., 2018). The detailed molecular mechanisms by which RipE modulates ATP levels and supports extracellular survival of *E. japonica* remain to be studied.

Despite the report of targeted mutagenesis (Hove et al., 2022) and genetic complementation (Wang et al., 2017) for *E. chaffeensis*, and functional trans-complementation for *E. japonica* (Zhang et al., 2024), restoration of *in vitro* and *in vivo* biological functions of mutated genes has been challenging. By transforming Δ*ripE* and WT *E. japonica* with another newly constructed Himar1 plasmid encoding gentamicin resistance and *ripE* genes (**Figure 6A**), we successfully created genetically complemented/rescued Δ*ripE* (Δ*ripE* ^+^) and RipE-overexpressing (WT^+^) *E. japonica*. Genomic complementation of the Δ*ripE* mutant partially restored *Ehrlichia* ATP level, and overexpression of *ripE* increased *Ehrlichia* ATP level. WT^+^-challenged mice succumbed to fatal ehrlichiosis much earlier than the WT, suggesting that RipE overexpression promotes *Ehrlichia in vivo* infection. The expression level of RipE protein in Δ*ripE* ^+^ *E. japonica* was, however, relatively low. It is known that genetic insertion by Himar1 transposon usually occurs once per genome or rarely twice (Lampe et al., 1999; Felsheim et al., 2006; Le Breton et al., 2006; Cain et al., 2020). Himar1 insertion not only alters gene functions by intragenic insertion of the transposon but also may disrupt important regulatory elements of the genome (Lampe et al., 1999; Munderloh et al., 2012), resulting in either a lethal phenotype lost during bacterial isolation or a transformed bacterium that cannot survive alone. Δ*ripE* ^+^ *E. japonica* contains at least two Himar1 insertions, including the original insertion disrupting *ripE* and the second one disrupting *mutS* gene. It is possible that the double Himar1 insertions resulted in the instability of some essential genes for *ripE* expression. While the current work demonstrates significant progress, the molecular complementation methods of obligatory intracellular bacteria remain to be improved at multiple fronts.

Considering RipE functions, it is peculiar why RipE is required only in *in vivo* infection of *E. japonica*, but not in cell culture. *Ehrlichia* species are fastidious and infect only specific types of host cells, such as monocytes/macrophages (Rikihisa, 1991). For cell culture, as we use overabundant host cells such as DH82 canine macrophages that are permissive to *Ehrlichia* infection, *Ehrlichia* has a short extracellular stage to initiate a new cycle of infection. However, *in vivo*, especially in the circulating blood of a mammalian host, these permissive blood monocytes are much less; thus, *Ehrlichia* needs to be extracellular for a relatively longer period before infecting next host cells. This may be one of the reasons why RipE is required *in vivo* for effective *Ehrlichia* infection.

The host cell-free *Ehrlichia* in this study were isolated by sonication and filtration, kept on ice, and subsequently used for *in vitro* and *in vivo* (i.v. inoculation) experiments immediately (within 30 min) to minimize the loss of viability and infectivity of these extracellular *Ehrlichia*. Our results showed that host cell-free *Ehrlichia* are capable of replicating in the mouse peripheral blood within 24 h following i.v. inoculation (**Figure 3C**). Li and Winslow (2003) reported that extracellular *Ehrlichia* not only can survive in the mouse plasma but also have limited ability to proliferate outside host cells. Host cell-free *E. japonica* preincubated in medium containing 50% NMP showed significantly higher infectivity compared to 50% heat-inactivated NMP, 50% heat-inactivated FBS, or DMEM. *Ehrlichia* has a highly fragile membrane that lacks LPS and peptidoglycan (Lin and Rikihisa, 2003). The presence of serum in the medium could physically protect the fragile extracellular *Ehrlichia*. *Ehrlichia* is known to incorporate cholesterol and other lipids (Lin and Rikihisa, 2003; Lin et al., 2020) and metabolize l-glutamine (Rikihisa et al., 1994; Lin et al., 2016). It is possible that lipids, lipoproteins, amino acids, vitamins, or other nutrients in the mammalian plasma/serum can support extracellular *Ehrlichia* survival. Heat inactivation (56°C, 30 min) denatures or inactivates not only complements, but also some of these molecules in the plasma (Pellerin et al., 2021; Zhang et al., 2024), resulting in reduced infectivity of extracellular *Ehrlichia.*

*E. chaffeensis* is spontaneously cleared by the immune system of immunocompetent mice and does not cause any significant clinical signs or mortalities. The current study is the first to demonstrate that immunization can delay the fatal ehrlichiosis caused by *E. japonica*, a highly virulent bacterium in mice. Of note, rRipE immunization significantly reduced bacteremia on day 5 post-challenge, but not at the later stage (day 8 post-challenge) when challenged with a high lethal dosage of ~10,000 *E. japonica* (**Figure 11C**). With respect to relationships between bacteremia and clinical disease, however, our previous study showed that clinical disease is related to tissue macrophage infection, rather than peripheral blood monocyte infection (Zhang et al., 2024). For the rRipE immunization and *E. japonica* challenge study, we aim to establish survival curves; thus, mice were kept until they succumbed to fatal ehrlichiosis, and therefore, comparable tissues/organs from the same time point (e.g., day 8 post-challenge) were not available for analysis in this case. On the other hand, for the *E. chaffeensis* challenge, *Ehrlichia* loads were significantly lower in blood and tissue samples collected on day 5 post-challenge.

*E. japonica* is highly virulent to laboratory mice, and the infection with ~5,000–11,000 bacteria (~50- to 100-fold of LD_50_ of 100 bacteria per mouse) results in death within 7–9 days after the i.p. challenge (**Figures 8D**, **11C**) (Bekebrede et al., 2020). It is unlikely that a detectable antibody titer against RipE or other *E. japonica* antigens is developed during this short period. Luo et al. reported that HME patient serum and sera from dogs with chronic *E. canis* infection also do not develop specific antibodies against RipE homologous proteins, ECH_0079 of *E. chaffeensis* and Ecaj_0047 of *E. canis*, respectively (Luo et al., 2020). This suggests that RipE-specific humoral immune response is not elicited during natural infections. On the other hand, our data showed that rRipE is immunogenic and capable of inducing a robust humoral response and *Ehrlichia*-specific neutralizing antibody in mice. Previous studies have shown that mice and dogs immunized with recombinant P28 or OMP-1B protein (major outer-membrane proteins), VirB2 (type IV secretion pilus protein), or EtpE (entry-triggering protein of *Ehrlichia*) of *E. chaffeensis* are protected from *E. chaffeensis* infection (Ohashi et al., 1998; Budachetri et al., 2020, Budachetri et al., 2022). rRipE immunization cross-protected mice from *E. chaffeensis* infection; thus, RipE can be an additional vaccine candidate against *E. chaffeensis* infection. The current study also poses challenges in developing *Ehrlichia* vaccines. Single-target vaccines may not be highly effective against *Ehrlichia* species as these obligate intracellular pathogens possess multiple virulence factors that compensate for the reduced ability of extracellular survival and help them evade host immunity (Yan et al., 2018; Budachetri et al., 2020; Rikihisa, 2021; Zhang et al., 2021; Budachetri et al., 2022). Therefore, a multivalent vaccine targeting multiple key virulence factors is expected to have a higher chance of success.

## Materials and methods

### Ethics statement for laboratory animal use

Animal experiments in this study were performed under approved protocols in accordance with guidelines of the Institutional Animal Care and Use Committee of the Ohio State University (OSU). The OSU has continued accreditation by the Association for Assessment and Accreditation of Laboratory Animal Care International, Animal Welfare Assurance from Public Health Services, United States Department of Agriculture, and is in full compliance with the PHS Policy and the Federal Animal Welfare Regulations.

### Protein sequence analysis and structural prediction

The *E. japonica* RipE protein sequence was analyzed by the Protean program (DNASTAR Lasergene) (Plasterer, 2000), and the three-dimensional structure diagram was rendered by the AlphaFold algorithm (https://alphafold.ebi.ac.uk/entry/X5H2X1) (Jumper et al., 2021). DeepLocPro (https://ku.biolib.com/deeplocpro) was used to predict the protein localization.

### Cloning, antibodies, and immunofluorescence assay

The gene encoding full-length *ripE* (EHF_0962) was PCR amplified using primers shown in **Supplementary Table S1** and cloned into pET33b(+) (Novagen, Gibbstown, NJ) for expressing rRipE with 6×His-tagged on both N- and C-terminus (rRipE). rRipE protein was affinity purified from soluble fractions in transformed *Escherichia coli* BL21(DE3) (New England Biolabs, Ipswich, MA) using HisPur Cobalt Resin (Thermo Fisher) as previously described (Kumagai et al., 2006). Mouse antiserum against rRipE was developed by immunizing C57BL/6 mice (Envigo, Indianapolis, IN) three times with subcutaneous injection of rRipE (100 µg per mouse) admixed with 10 µg of Quil-A adjuvant (InvivoGen, San Diego, CA) at 2-week intervals. Other antibodies used in this study include rabbit anti-*Ehrlichia* P28 (Ohashi et al., 1998), rabbit anti-β-actin (Sigma-Aldrich, St. Louis, MO), and Alexa Fluor (AF) 488- or AF555-conjugated goat anti-mouse or anti-rabbit IgG (Invitrogen, Carlsbad, CA).

For immunofluorescence assay, *Ehrlichia*-infected DH82 or RF/6A cells or host cell-free *Ehrlichia* were fixed with 4% paraformaldehyde (PFA), washed, and incubated with primary antibodies and then fluorescence-conjugated secondary antibodies diluted in PGS permeabilization buffer (PBS with 0.1% gelatin, 0.05% BSA, and 0.3% saponin) or nonpermeable blocking buffer (PBS with 0.1% gelatin and 0.05% BSA). DAPI (4′,6′-diamidino-2-phenylindole, Invitrogen) or the cell-permeant Hoechst 33342 (Invitrogen) was used to stain nucleic acid. Fluorescence with overlay differential interference contrast (DIC) images were acquired with a DeltaVision PersonalDV deconvolution microscope system (GE Healthcare Life Sciences, Marlborough, MA) or a THUNDER imaging system with computational clearing to remove out-of-focus blur background (Leica Microsystems, Deerfield, IL). Image analysis was performed with ImageJ software (National Institutes of Health, Bethesda, MD). Antibodies used and dilutions are summarized in **Supplementary Table S2**.

### Culture and purification of *E. japonica*, and separation of membrane fractions

The canine histiocytic leukemia cell line DH82 (Wellman et al., 1988) was cultured in DMEM (Dulbecco’s Modified Eagle’s Medium; Corning, Corning, NY) supplemented with 4% FBS (Atlanta Biologicals, Lawrenceville, GA) and 4 mM l-glutamine (Gln, Gibco, Waltham, MA) in a humidified incubator at 37°C with 5% CO_2_. The monkey endothelial cell line RF/6A was cultured in advanced MEM (Corning) with 8% FBS and 4 mM l-Gln. *E. chaffeensis* Arkansas strain (Dawson et al., 1991) was cultured in THP-1 cells (ATCC, Manassas, VA) (Barnewall and Rikihisa, 1994) in RPMI 1640 medium (Mediatech, Manassas, VA) supplemented with 8% FBS and 2 mM l-Gln.

*E. japonica*, Δ*ripE*, and other Himar1 mutants were cultured in DH82 cells with medium supplemented with 4% FBS, 4 mM Gln, and 0.1 µg/mL cycloheximide (MilliporeSigma, Burlington, MA) (Bekebrede et al., 2020). *Ehrlichia* infectivities were examined by HEMA3 staining (Thermo Fisher, Waltham, MA) of cytocentrifuged slides using Shandon CytoSpin 4 centrifuge (Thermo Fisher). Host cell-free *E. japonica* was purified from highly infected DH82 cells by sonication on ice for 8 s twice at output setting 2 using a fine tip on a W380 Sonicator (Heat Systems, Newtown, CT). Following centrifugation at 700 × *g* to remove unbroken host cells and nuclei, host cell-free bacteria were pelleted at 10,000 × *g*. To obtain *Ehrlichia* outer-membrane fractions, host cell-free *E. japonica* was solubilized with 0.1% Sarkosyl, and outer-membrane fractions were collected by ultracentrifugation as previously described (Ohashi et al., 1998). The osmotic shock method was used for extraction of bacterial periplasm and membrane fractions as previously described (Cheon et al., 2021).

Synchronous culture of *Ehrlichia* was performed as previously described (Cheng et al., 2008; Liu et al., 2012). Briefly, sonicated infected cells were passed through a 2.7-µm syringe filter and subjected to a second round of strong sonication on ice (30 s, twice, at output setting 4.5) to destroy RC forms. The remaining viable DC forms were pelleted and incubated with DH82 cells at 100 multiplicity of infection (MOI) at 37°C for 1 h with periodic shaking, followed by washing to remove uninternalized bacteria.

The growth curves of *Ehrlichia* in RF/6A cells were conducted by incubating host cell-free *Ehrlichia* with RF/6A cells at 300–400 MOI in a 24-well plate for 2 h at 37°C, followed by PBS washing to remove unbound *Ehrlichia* and culturing in RF/6A medium. Infected RF/6A cells were harvested at 0, 1, 3, and 5 dpi for DNA extraction and gene-specific qPCR as described below.

### PCR, qPCR, and RT-qPCR

DNA and RNA samples were purified using the DNeasy Blood & Tissue Kit (Qiagen) and the RNeasy Mini Kit (Qiagen), respectively, according to the manufacturer’s instructions. Flanking PCR for Δ*ripE* clonality verification was performed with gene-specific primers (**Supplementary Table S1**) as previously described (Bekebrede et al., 2020). cDNA was synthesized from 0.5 to 1 µg of RNA using the Maxima H Minus First Strand cDNA Synthesis Kit with random hexamer primers (Thermo Fisher). qPCR and RT-qPCR analysis were performed using Maxima SYBR Green/ROX Master Mix (Thermo Fisher) according to the manufacturer’s protocols in Mx3005P (Stratagene, Waltham, MA) or the AriaMx Real-Time PCR System (Agilent, Santa Clara, CA).

### *In vitro* infectivity, binding, and neutralization assay

To determine the binding, internalization, and infectivity of WT or Δ*ripE E. japonica*, host cell-free bacteria were preincubated in various media or antisera at 37°C for 30–60 min, including 50% NMP diluted in PBS with or without heat inactivation at 56°C for 30 min, 50% heat-inactivated FBS, serum-free DMEM, or neutralizing antisera against RipE or *Ehrlichia* P28. Treated *Ehrlichia* was incubated with RF/6A or DH82 cells, and the bacterial infectivity was determined at 2 dpi by *Ehrlichia* 16S rRNA-specific qPCR. To examine bound or internalized *E. japonica*, two rounds of immunostaining with anti-P28 were performed at 2 h pi as described previously (Mohan Kumar et al., 2013). Briefly, to detect surface-bound *Ehrlichia*, PFA-fixed cells were first labeled with anti-P28 and AF488-conjugated secondary antibody diluted in nonpermeable buffer. To detect internalized *Ehrlichia*, the second round of staining was performed with anti-P28 and AF555-conjugated secondary antibody diluted in PGS buffer.

As obligate intracellular bacteria, *Ehrlichia* spp. gradually lose infectivity when released from the infected host cells and thus need to be used immediately for both *in vitro* and *in vivo* experiments following purification. Aliquots of the inoculum (*Ehrlichia*-infected cell culture or host cell-free *Ehrlichia*) were saved for DNA extraction and qPCR, and the absolute bacteria inoculum was quantified by qPCR using *Ehrlichia* 16S rDNA cloned into pUC19 plasmids as standards (Teymournejad et al., 2017). Therefore, the exact MOI used in the experiments was determined after the inoculation of cells or mouse infection (Bekebrede et al., 2020), causing inoculum variations among different experiments.

### Mouse studies

Several mouse strains have been initially used for *E. japonica*, aka IOE infection (Fujita, 1994). The most frequently used strains include ddY, C57BL/6, and ICR, which show similar clinical signs with fatal *E. japonica* infection (Shibata et al., 2000; Sotomayor et al., 2001; Okada et al., 2003; Bekebrede et al., 2020). We previously established and demonstrated a dose-dependent infection of *E. japonica* in ICR mice with an LD_50_ of 100 bacteria per mouse, which well recapitulates the full spectrum of human ehrlichiosis (Bekebrede et al., 2020). In this study, C57BL/6 mice were used in the time-course experiments to compare the infection of WT vs. Δ*ripE E. japonica* as C57BL/6 are inbred mice, which will likely minimize the individual mouse differences and maximize the potential difference/significance directly related to the *ripE* gene in the infected mouse. ICR mice are outbred strain, which represents a population of heterogenous genome background and is used mainly for the immunization (with rRipE) experiments in this study to demonstrate the immune response and protectivity of rRipE immunization in a relatively diverse population, mimicking the human populations.

C57BL/6 mice were originally from Envigo (currently Inotiv; West Lafayette, IN) and bred at OSU animal facility. ICR mice were purchased from Inotiv and used at 4–8 weeks old. Mice were i.p. or retro-orbitally (intravenous, i.v.) (Yardeni et al., 2011) injected with WT, Δ*ripE*, ΔripE^+^, or WT^+^ *E. japonica*-infected DH82 cells. To test whether rRipE immunization can protect the mouse from fatal ehrlichiosis caused by *E. japonica*, ICR mice were first immunized with rRipE as described earlier or sham-immunized with Quil-A only and challenged with i.p. inoculation of ~11,750 WT *E. japonica* at 9 days post third immunization. Mice were weighted and monitored for clinical signs daily. For the *E. japonica* challenge, a small volume (20–50 µL) of blood samples was collected through facial vein phlebotomy at the time points shown in each figure for *Ehrlichia* 16S qPCR to determine the bacteria loads.

To test whether rRipE immunization can protect mice infection from human ehrlichiosis agent *E. chaffeensis*, C57BL/6 mice were immunized with rRipE as described above and challenged at 11 days post third immunization with i.p. inoculation of infected THP-1 cells containing ~115,000 *E. chaffeensis* for 5 days. Mice were euthanized by CO_2_ inhalation followed by cervical dislocation, and the resident peritoneal cells were collected by rinsing the peritoneal cavity with 5–10 mL of sterile PBS containing 2 mM sodium EDTA for at least three times. Blood, liver, and spleen samples were also harvested and stored at –20°C for DNA extraction, or preserved in RNAlater buffer (Qiagen, Germantown, MD) and stored at –80°C until RNA extraction.

### ELISA and Western blot analysis

The wells of a 96-well flat-bottom microtiter plate (Nunc MaxiSorp, MilliporeSigma) were coated with 1 μg of rRipE or BSA, and ELISA analyses of sera from sham-vaccinated or vaccinated mice were performed as previously described (Budachetri et al., 2020). For Western blot analysis, *Ehrlichia*-infected DH82 cells were lysed in RIPA buffer (150 mM NaCl, 25 mM Tris-HCl, 1% Triton X-100, and 0.5% w/v sodium deoxycholate) with freshly added 1× Protease Inhibitor Cocktail (MilliporeSigma). The cell lysates and rRipE were subject to Western blotting using primary antibodies and horseradish peroxidase-conjugated secondary antibodies (SeraCare, Milford, MA). Mouse antisera against rRipE were pre-adsorbed with uninfected DH82 cell lysate transferred onto a nitrocellulose membrane (Bio-Rad, Hercules, CA) to reduce nonspecific binding. Reacting bands were visualized with Pierce ECL Western Blotting Substrate (Thermo Fisher), and images were captured and quantitated by the Amersham AI680QC gel documentation system (GE Healthcare, Marlborough, MA).

### Luminescent ATP detection assay

ATP levels were determined by using the Luminescent ATP Detection Assay Kit (Abcam, Cambridge, UK) according to the manufacturer’s instructions. Briefly, host cell-free *Ehrlichia* and dilutions of ATP standards in DMEM were transferred into a white, flat-bottom 96-well plate (BrandTech, Wertheim, Germany), and the detergent component was added to assist cell lysis and ATP stabilization. For luminescence development, substrate solution was added and incubated for 10 min, and the ATP levels were measured by a Synergy HTX multimode reader (BioTek, Winooski, VT).

### Genomic complementation and overexpression of *ripE*

pCis-FLAG-RipE-Gent-Himar plasmid was constructed by replacing Himar1 insertion sequences flanked by 5′- and 3′-inverted terminal repeats (ITRs) in the pCis-FLAG-Etf-2-SS-Himar A7 plasmid (Cheng et al., 2013; Yan et al., 2018) with *E. japonica ripE* and gentamicin resistance (*Gent*) genes. *Gent* gene was amplified from pGGA-Tuf-Cherry-Gent plasmids (**Supplementary Table S1**) (Hove et al., 2022). The resulting plasmid encodes FLAG-*ripE* and *Gent* genes with polycistronic expression driven by the *Anaplasma marginale* transcriptional regulator 1 (Tr1) promoter (**Figure 6A**). Plasmids were transformed into *dam* ^–^/*dcm* ^–^ competent *E. coli* (New England Biolabs) and purified using the EndoFree Maxiprep Plasmid Kit (Qiagen).

*E. japonica* transformation was performed as described previously with minor modifications (Yan et al., 2018). Briefly, host cell-free *E. japonica* was resuspended in 300 mM sucrose solution, mixed with 10–15 µg of plasmid in a 0.2-cm gap cuvette (Bio-Rad), and electroporated at 2,500 V, 25 µF, and 400 Ω using a Gene Pulser Xcell Electroporation System (Bio-Rad). The transformed *E. japonica* was used to infect DH82 cells for 2 days and selected in DMEM medium containing 1% FBS, 4 mM l-Gln, 0.1 µg/mL cycloheximide, and 20 µg/mL gentamicin. The culture medium was replaced two to three times a week until the infection rate reached ≥80% in 2–4 weeks. Successfully transformed *E. japonica* became visible on cytocentrifuged slides within 17 to 31 days pi. The new insertion site in the transformed *E. japonica* was determined using semi-random, two-step PCR (ST-PCR) and sequencing as previously described (Cheng et al., 2013; Bekebrede et al., 2020) and then verified by insertion site-specific flanking PCR primers (**Supplementary Table S1**).

### Statistical analysis

All statistical analyses were performed using GraphPad Prism 9 (GraphPad Software, Boston, MA). For data with only one time point, unpaired Student’s *t*-test or one-way analysis of variance (ANOVA) with Tukey’s multiple comparisons was used. Data with multiple time points were analyzed with repeated-measures ANOVA and Šídák’s multiple comparisons test. Kaplan–Meier survival curves were analyzed by log-rank (Mantel–Cox) test. In all tests, *p* < 0.05 was considered statistically significant.

## Data Availability

The original contributions presented in the study are included in the article/**Supplementary Material**. Further inquiries can be directed to the corresponding author.

## References

[B1] Ahmed I. Ismail N. (2020). M1 and M2 Macrophages Polarization via mTORC1 Influences Innate Immunity and Outcome of Ehrlichia Infection. J. Cell Immunol. 2, 108–115. doi: 10.33696/immunology.2.029 32719831 PMC7384756

[B2] Bakken J. S. Dumler J. S. (2000). Human granulocytic ehrlichiosis. Clin. Infect. Dis. 31, 554–560. doi: 10.1086/313948 10987720

[B3] Barnewall R. E. Rikihisa Y. (1994). Abrogation of gamma interferon-induced inhibition of *Ehrlichia chaffeensis* infection in human monocytes with iron-transferrin. Infect. Immun. 62, 4804–4810. doi: 10.1128/iai.62.11.4804-4810.1994 7927758 PMC303190

[B4] Bekebrede H. Lin M. Teymournejad O. Rikihisa Y. (2020). Discovery of *in vivo* virulence genes of obligatory intracellular bacteria by random mutagenesis. Front. Cell Infect. Microbiol. 10. doi: 10.3389/fcimb.2020.00002 PMC7010607 32117791

[B5] Budachetri K. Lin M. Chien R. C. Zhang W. Brock G. N. Rikihisa Y. (2022). Efficacy and immune correlates of OMP-1B and virB2-4 vaccines for protection of dogs from tick transmission of ehrlichia chaffeensis. mBio 13, e0214022. doi: 10.1128/mbio.02140-22 36342170 PMC9765013

[B6] Budachetri K. Teymournejad O. Lin M. Yan Q. Mestres-Villanueva M. Brock G. N.. (2020). An entry-triggering protein of ehrlichia is a new vaccine candidate against tick-borne human monocytic ehrlichiosis. mBio 11, e00895-20. doi: 10.1128/mBio.00895-20 32723916 PMC7387794

[B7] Cain A. K. Barquist L. Goodman A. L. Paulsen I. T. Parkhill J. van Opijnen T. (2020). A decade of advances in transposon-insertion sequencing. Nat. Rev. Genet. 21, 526–540. doi: 10.1038/s41576-020-0244-x 32533119 PMC7291929

[B8] Cartman S. T. Minton N. P. (2010). A mariner-based transposon system for *in vivo* random mutagenesis of Clostridium difficile. Appl. Environ. Microbiol. 76, 1103–1109. doi: 10.1128/AEM.02525-09 20023081 PMC2820977

[B9] CDC (2024). National Notifiable Diseases Surveillance System, Weekly Tables of Infectious Disease Data (Atlanta, GA: Centers for Disease Control and Prevention, Division of Health Informatics and Surveillance).

[B10] Cheng Z. Miura K. Popov V. L. Kumagai Y. Rikihisa Y. (2011). Insights into the CtrA regulon in development of stress resistance in obligatory intracellular pathogen *Ehrlichia chaffeensis* . Mol. Microbiol. 82, 1217–1234. doi: 10.1111/j.1365-2958.2011.07885.x 22014113 PMC3241975

[B11] Cheng C. Nair A. D. Indukuri V. V. Gong S. Felsheim R. F. Jaworski D.. (2013). Targeted and random mutagenesis of *Ehrlichia chaffeensis* for the identification of genes required for *in vivo* infection. PLoS Pathog. 9, e1003171. doi: 10.1371/journal.ppat.1003171 23459099 PMC3573109

[B12] Cheng Z. Wang X. Rikihisa Y. (2008). Regulation of type IV secretion apparatus genes during *Ehrlichia chaffeensis* intracellular development by a previously unidentified protein. J. Bacteriol 190, 2096–2105. doi: 10.1128/JB.01813-07 18192398 PMC2258868

[B13] Cheon D. H. Lee S. Yang W. S. Hwang S. Jang H. Kim M. J.. (2021). Optimization of a lysis method to isolate periplasmic proteins from Gram-negative bacteria for clinical mass spectrometry. Proteomics Clin. Appl. 15, e2100044. doi: 10.1002/prca.202100044 34370896

[B14] Cossé M. M. Hayward R. D. Subtil A. (2018). One face of chlamydia trachomatis: The infectious elementary body. Curr. Top. Microbiol. Immunol. 412, 35–58. doi: 10.1007/82_2016_12 27197644

[B15] Dawson J. E. Anderson B. E. Fishbein D. B. Sanchez J. L. Goldsmith C. S. Wilson K. H.. (1991). Isolation and characterization of an Ehrlichia sp. from a patient diagnosed with human ehrlichiosis. J. Clin. Microbiol. 29, 2741–2745. doi: 10.1128/jcm.29.12.2741-2745.1991 1757543 PMC270425

[B16] Dumler J. S. Barbet A. F. Bekker C. P. Dasch G. A. Palmer G. H. Ray S. C.. (2001). Reorganization of genera in the families Rickettsiaceae and Anaplasmataceae in the order Rickettsiales: unification of some species of Ehrlichia with Anaplasma, Cowdria with Ehrlichia and Ehrlichia with Neorickettsia, descriptions of six new species combinations and designation of Ehrlichia equi and 'HGE agent' as subjective synonyms of Ehrlichia phagocytophila. Int. J. Syst. Evol. Microbiol. 51, 2145–2165. doi: 10.1099/00207713-51-6-2145 11760958

[B17] Dunning Hotopp J. C. Lin M. Madupu R. Crabtree J. Angiuoli S. V. Eisen J.. (2006). Comparative genomics of emerging human ehrlichiosis agents. PLoS Genet. 2, e21. doi: 10.1371/journal.pgen.0020021 16482227 PMC1366493

[B18] Eedunuri V. K. Zhang Y. Cheng C. Chen L. Liu H. Omsland A.. (2018). Protein and DNA synthesis demonstrated in cell-free Ehrlichia chaffeensis organisms in axenic medium. Sci. Rep. 8, 9293. doi: 10.1038/s41598-018-27574-z 29915240 PMC6006305

[B19] Felsheim R. F. Herron M. J. Nelson C. M. Burkhardt N. Y. Barbet A. F. Kurtti T. J.. (2006). Transformation of anaplasma phagocytophilum. BMC Biotechnol. 6, 42. doi: 10.1186/1472-6750-6-42 17076894 PMC1635035

[B20] Fujita H. (1994). Ehrlichial organisms isolated from Ixodes ovatus ticks and field rodents in Japan. Ann. Rep. Ohara Hosp. 37, 13–17.

[B21] Habib S. El Andaloussi A. Hisham A. Ismail N. (2016). NK cell-mediated regulation of protective memory responses against intracellular ehrlichial pathogens. PLoS One 11, e0153223. doi: 10.1371/journal.pone.0153223 27092553 PMC4836677

[B22] Haloul M. Oliveira E. R. A. Kader M. Wells J. Z. Tominello T. R. El Andaloussi A.. (2019). mTORC1-mediated polarization of M1 macrophages and their accumulation in the liver correlate with immunopathology in fatal ehrlichiosis. Sci. Rep. 9, 14050. doi: 10.1038/s41598-019-50320-y 31575880 PMC6773708

[B23] Hove P. Madesh S. Nair A. Jaworski D. Liu H. Ferm J.. (2022). Targeted mutagenesis in Anaplasma marginale to define virulence and vaccine development against bovine anaplasmosis. PLoS Pathog. 18, e1010540. doi: 10.1371/journal.ppat.1010540 35576225 PMC9135337

[B24] Ismail N. Sharma A. Soong L. Walker D. H. (2022). Review: protective immunity and immunopathology of ehrlichiosis. Zoonoses (Burlingt) 2. doi: 10.15212/zoonoses-2022-0009 PMC9300479 35876763

[B25] Ismail N. Stevenson H. L. Walker D. H. (2006). Role of tumor necrosis factor alpha (TNF-alpha) and interleukin-10 in the pathogenesis of severe murine monocytotropic ehrlichiosis: increased resistance of TNF receptor p55- and p75-deficient mice to fatal ehrlichial infection. Infect. Immun. 74, 1846–1856. doi: 10.1128/IAI.74.3.1846-1856.2006 16495559 PMC1418656

[B26] Izadi-Pruneyre N. Wolff N. Redeker V. Wandersman C. Delepierre M. Lecroisey A. (1999). NMR studies of the C-terminal secretion signal of the haem-binding protein, HasA. Eur. J. Biochem. 261, 562–568. doi: 10.1046/j.1432-1327.1999.00305.x 10215870

[B27] Jumper J. Evans R. Pritzel A. Green T. Figurnov M. Ronneberger O.. (2021). Highly accurate protein structure prediction with AlphaFold. Nature 596, 583–589. doi: 10.1038/s41586-021-03819-2 34265844 PMC8371605

[B28] Kumagai Y. Cheng Z. Lin M. Rikihisa Y. (2006). Biochemical activities of three pairs of *Ehrlichia chaffeensis* two-component regulatory system proteins involved in inhibition of lysosomal fusion. Infect. Immun. 74, 5014–5022. doi: 10.1128/IAI.00735-06 16926392 PMC1594868

[B29] Kumagai Y. Matsuo J. Hayakawa Y. Rikihisa Y. (2010). Cyclic di-GMP signaling regulates invasion by *Ehrlichia chaffeensis* of human monocytes. J. Bacteriol 192, 4122–4133. doi: 10.1128/JB.00132-10 20562302 PMC2916414

[B30] Lampe D. J. Akerley B. J. Rubin E. J. Mekalanos J. J. Robertson H. M. (1999). Hyperactive transposase mutants of the Himar1 mariner transposon. Proc. Natl. Acad. Sci. U.S.A. 96, 11428–11433. doi: 10.1073/pnas.96.20.11428 10500193 PMC18050

[B31] Le Breton Y. Mohapatra N. P. Haldenwang W. G. (2006). *In vivo* random mutagenesis of Bacillus subtilis by use of TnYLB-1, a mariner-based transposon. Appl. Environ. Microbiol. 72, 327–333. doi: 10.1128/Aem.72.1.327-333.2006 16391061 PMC1352254

[B32] Li J. S. Winslow G. M.. (2003). Survival, replication, and antibody susceptibility of Ehrlichia chaffeensis outside of host cells. Infect Immun. 71, 4229–37. doi: 10.1128/IAI.71.8.4229-4237.2003 12874298 PMC166042

[B33] Li J. S. Yager E. Reilly M. Freeman C. Reddy G. R. Reilly A. A.. (2001). Outer membrane protein-specific monoclonal antibodies protect SCID mice from fatal infection by the obligate intracellular bacterial pathogen *Ehrlichia chaffeensis* . J. Immunol. 166, 1855–1862. doi: 10.4049/jimmunol.166.3.1855 11160232

[B34] Lin M. Grandinetti G. Hartnell L. M. Bliss D. Subramaniam S. Rikihisa Y. (2020). Host membrane lipids are trafficked to membranes of intravacuolar bacterium Ehrlichia chaffeensis. Proc. Natl. Acad. Sci. U.S.A. 117, 8032–8043. doi: 10.1073/pnas.1921619117 32193339 PMC7149431

[B35] Lin M. Liu H. Xiong Q. Niu H. Cheng Z. Yamamoto A.. (2016). Ehrlichia secretes Etf-1 to induce autophagy and capture nutrients for its growth through RAB5 and class III phosphatidylinositol 3-kinase. Autophagy 12, 2145–2166. doi: 10.1080/15548627.2016.1217369 27541856 PMC5103349

[B36] Lin M. Rikihisa Y. (2003). *Ehrlichia chaffeensis* and *Anaplasma phagocytophilum* lack genes for lipid A biosynthesis and incorporate cholesterol for their survival. Infect. Immun. 71, 5324–5331. doi: 10.1128/IAI.71.9.5324-5331.2003 12933880 PMC187327

[B37] Lin M. Xiong Q. Chung M. Daugherty S. C. Nagaraj S. Sengamalay N.. (2021). Comparative analysis of genome of ehrlichia sp. HF, a model bacterium to study fatal human ehrlichiosis. BMC Genomics 22, 11. doi: 10.1186/s12864-020-07309-z 33407096 PMC7789307

[B38] Liu H. Bao W. Lin M. Niu H. Rikihisa Y. (2012). Ehrlichia type IV secretion effector ECH0825 is translocated to mitochondria and curbs ROS and apoptosis by upregulating host MnSOD. Cell Microbiol. 14, 1037–1050. doi: 10.1111/j.1462-5822.2012.01775.x 22348527 PMC3371182

[B39] Luo T. Patel J. G. Zhang X. Walker D. H. McBride J. W. (2020). Ehrlichia chaffeensis and E. canis hypothetical protein immunoanalysis reveals small secreted immunodominant proteins and conformation-dependent antibody epitopes. NPJ Vaccines 5, 85. doi: 10.1038/s41541-020-00231-1 32963815 PMC7486380

[B40] McGill J. L. Nair A. D. Cheng C. Rusk R. A. Jaworski D. C. Ganta R. R. (2016). Vaccination with an attenuated mutant of ehrlichia chaffeensis induces pathogen-specific CD4+ T cell immunity and protection from tick-transmitted wild-type challenge in the canine host. PLoS One 11, e0148229. doi: 10.1371/journal.pone.0148229 26841025 PMC4739596

[B41] Mohan Kumar D. Yamaguchi M. Miura K. Lin M. Los M. Coy J. F.. (2013). *Ehrlichia chaffeensis* uses its surface protein EtpE to bind GPI-anchored protein DNase X and trigger entry into mammalian cells. PLoS Pathog. 9, e1003666. doi: 10.1371/journal.ppat.1003666 24098122 PMC3789761

[B42] Moreno J. Nielsen H. Winther O. Teufel F. (2024). Predicting the subcellular location of prokaryotic proteins with DeepLocPro. bioRxiv 2024, 2001.2004.574157. doi: 10.1101/2024.01.04.574157 PMC11645106 39540738

[B43] Munderloh U. Burkhardt N. Herron M. Oliva Chavez A. Nelson C. Kurtti T. (2012). “Intracellular pathogens II: rickettsiales,” in The Way Forward: Improving Genetic Systems. (ASM Press New York, NY), 271–272.

[B44] Ohashi N. Zhi N. Zhang Y. Rikihisa Y. (1998). Immunodominant major outer membrane proteins of *Ehrlichia chaffeensis* are encoded by a polymorphic multigene family. Infect. Immun. 66, 132–139. doi: 10.1128/IAI.66.1.132-139.1998 9423849 PMC107868

[B45] Okada H. Tajima T. Kawahara M. Rikihisa Y. (2001). Ehrlichial proliferation and acute hepatocellular necrosis in immunocompetent mice experimentally infected with the HF strain of *Ehrlichia*, closely related to *Ehrlichia chaffeensis* . J. Comp. Pathol. 124, 165–171. doi: 10.1053/jcpa.2000.0447 11222014

[B46] Okada H. Usuda H. Tajima T. Kawahara M. Yoshino T. Rikihisa Y. (2003). Distribution of ehrlichiae in tissues as determined by *in-situ* hybridization. J. Comp. Pathol. 128, 182–187. doi: 10.1053/jcpa.2002.0624 12634096

[B47] Omsland A. Sager J. Nair V. Sturdevant D. E. Hackstadt T. (2012). Developmental stage-specific metabolic and transcriptional activity of Chlamydia trachomatis in an axenic medium. Proc. Natl. Acad. Sci. U.S.A. 109, 19781–19785. doi: 10.1073/pnas.1212831109 23129646 PMC3511728

[B48] Oren A. Garrity G. M. (2022). Validation List no. 206. Valid publication of new names and new combinations effectively published outside the IJSEM. Int. J. Syst. Evol. Microbiol. 72, 005422. doi: 10.1099/ijsem.0.005422 35904866

[B49] Pellerin F. A. Caneparo C. Pellerin E. Chabaud S. Pelletier M. Bolduc S. (2021). Heat-Inactivation of fetal and newborn sera did not impair the expansion and scaffold engineering potentials of fibroblasts. Bioengineering (Basel) 8, 184. doi: 10.3390/bioengineering8110184 34821750 PMC8615100

[B50] Plasterer T. N. (2000). PROTEAN. Protein sequence analysis and prediction. Mol. Biotechnol. 16, 117–125. doi: 10.1385/MB:16:2:117 11131972

[B51] Popov V. L. Chen S. M. Feng H. M. Walker D. H. (1995). Ultrastructural variation of cultured *Ehrlichia chaffeensis* . J. Med. Microbiol. 43, 411–421. doi: 10.1099/00222615-43-6-411 7473674

[B52] Rikihisa Y. (1991). The tribe *Ehrlichieae* and ehrlichial diseases. Clin. Microbiol. Rev. 4, 286–308. doi: 10.1128/CMR.4.3.286 1889044 PMC358200

[B53] Rikihisa Y. (2010). *Anaplasma phagocytophilum* and *Ehrlichia chaffeensis*: subversive manipulators of host cells. Nat. Rev. Microbiol. 8, 328–339. doi: 10.1038/nrmicro2318 20372158

[B54] Rikihisa Y. (2015). Molecular pathogenesis of *ehrlichia chaffeensis* infection. Annu. Rev. Microbiol. 69, 283–304. doi: 10.1146/annurev-micro-091014-104411 26488275

[B55] Rikihisa Y. (2021). The "Biological weapons" of ehrlichia chaffeensis: novel molecules and mechanisms to subjugate host cells. Front. Cell Infect. Microbiol. 11. doi: 10.3389/fcimb.2021.830180 PMC8834651 35155275

[B56] Rikihisa Y. Zhang Y. Park J. (1994). Inhibition of infection of macrophages with *Ehrlichia risticii* by cytochalasins, monodansylcadaverine, and taxol. Infect. Immun. 62, 5126–5132. doi: 10.1128/iai.62.11.5126-5132.1994 7927796 PMC303234

[B57] Riley S. P. Fish A. I. Del Piero F. Martinez J. J. (2018). Immunity against the Obligate Intracellular Bacterial Pathogen Rickettsia australis Requires a Functional Complement System. Infect. Immun. 86, e00139-18. doi: 10.1128/IAI.00139-18 29581196 PMC5964522

[B58] Sharma A. K. El Andaloussi A. Ismail N. (2023). Evasion of host antioxidative response via disruption of NRF2 signaling in fatal Ehrlichia-induced liver injury. PLoS Pathog. 19, e1011791. doi: 10.1371/journal.ppat.1011791 37956169 PMC10681308

[B59] Shibata S. Kawahara M. Rikihisa Y. Fujita H. Watanabe Y. Suto C.. (2000). New *Ehrlichia* species closely related to *Ehrlichia chaffeensis* isolated from *Ixodes ovatus* ticks in Japan. J. Clin. Microbiol. 38, 1331–1338. doi: 10.1128/JCM.38.4.1331-1338.2000 10747103 PMC86441

[B60] Skipp P. J. Hughes C. McKenna T. Edwards R. Langridge J. Thomson N. R.. (2016). Quantitative proteomics of the infectious and replicative forms of chlamydia trachomatis. PLoS One 11, e0149011. doi: 10.1371/journal.pone.0149011 26871455 PMC4752267

[B61] Sotomayor E. A. Popov V. L. Feng H. M. Walker D. H. Olano J. P. (2001). Animal model of fatal human monocytotropic ehrlichiosis. Am. J. Pathol. 158, 757–769. doi: 10.1016/S0002-9440(10)64018-7 11159213 PMC1850300

[B62] Teymournejad O. Lin M. Rikihisa Y. (2017). Ehrlichia chaffeensis and its invasin etpE block reactive oxygen species generation by macrophages in a DNase X-dependent manner. MBio 8, e01551-17. doi: 10.1128/mBio.01551-17 29162709 PMC5698551

[B63] Thomas S. (2016). Development of structure-based vaccines for ehrlichiosis. Methods Mol. Biol. 1403, 519–534. doi: 10.1007/978-1-4939-3387-7_29 27076151

[B64] Tipples G. McClarty G. (1993). The obligate intracellular bacterium Chlamydia trachomatis is auxotrophic for three of the four ribonucleoside triphosphates. Mol. Microbiol. 8, 1105–1114. doi: 10.1111/j.1365-2958.1993.tb01655.x 8361355

[B65] Wang Y. Wei L. Liu H. Cheng C. Ganta R. R. (2017). A genetic system for targeted mutations to disrupt and restore genes in the obligate bacterium, Ehrlichia chaffeensis. Sci. Rep. 7, 15801. doi: 10.1038/s41598-017-16023-y 29150636 PMC5693922

[B66] Weiss E. Williams J. C. Dasch G. A. Kang Y. H. (1989). Energy metabolism of monocytic Ehrlichia. Proc. Natl. Acad. Sci. U.S.A. 86, 1674–1678. doi: 10.1073/pnas.86.5.1674 2922404 PMC286763

[B67] Wellman M. L. Krakowka S. Jacobs R. M. Kociba G. J. (1988). A macrophage-monocyte cell line from a dog with Malignant histiocytosis. In Vitro Cell Dev. Biol. 24, 223–229. doi: 10.1007/BF02623551 3350786

[B68] Winslow G. M. Bitsaktsis C. Yager E. (2005). Susceptibility and resistance to monocytic ehrlichiosis in the mouse. Ann. N Y Acad. Sci. 1063, 395–402. doi: 10.1196/annals.1355.071 16481547

[B69] Yan Q. Lin M. Huang W. Teymournejad O. Johnson J. M. Hays F. A.. (2018). Ehrlichia type IV secretion system effector Etf-2 binds to active RAB5 and delays endosome maturation. Proc. Natl. Acad. Sci. U.S.A. 115, E8977–E8986. doi: 10.1073/pnas.1806904115 PMC6156607 30181274

[B70] Yardeni T. Eckhaus M. Morris H. D. Huizing M. Hoogstraten-Miller S. (2011). Retro-orbital injections in mice. Lab. Anim. (NY) 40, 155–160. doi: 10.1038/laban0511-155 21508954 PMC3158461

[B71] Zhang T. Chien R. C. Budachetri K. Lin M. Boyaka P. Huang W.. (2024). Ehrlichia effector TRP120 manipulates bacteremia to facilitate tick acquisition. mBio 15, e0047624. doi: 10.1128/mbio.00476-24 38501870 PMC11005420

[B72] Zhang W. Lin M. Yan Q. Budachetri K. Hou L. Sahni A.. (2021). An intracellular nanobody targeting T4SS effector inhibits Ehrlichia infection. Proc. Natl. Acad. Sci. U.S.A. 118, e2024102118. doi: 10.1073/pnas.2024102118 33903242 PMC8106314

[B73] Zhang J. Z. Popov V. L. Gao S. Walker D. H. Yu X. J. (2007). The developmental cycle of *Ehrlichia chaffeensis* in vertebrate cells. Cell Microbiol. 9, 610–618. doi: 10.1111/j.1462-5822.2006.00812.x 16987329

