# Supplementary material for: RipE expression correlates with high ATP levels in Ehrlichia, which confers resistance during the extracellular stage to facilitate a new cycle of infection
Source: Front Cell Infect Microbiol. 2024 Oct 1;14:1416577. doi: 10.3389/fcimb.2024.1416577 (PMC11473500; doi:10.3389/fcimb.2024.1416577)
Supplement: Supplementary file 1 [file DataSheet1.pdf]

Supplemental Tables

Table S1. Primers used in this study for cloning and qPCR

**Target** [1]	**Primer sequence** [2]	**Notes**
**Recombinant protein expression in *E. coli*:**		
ripE	F: GCCG CATATG ATAAGCACAAAAAACACTGACACAAC<br>R: GACT CTCGAG TTGTGTTGATGAAAAATACTCCTTGAGTG	Underlined: F, NdeI; R, XhoI.<br>*ripE* was cloned into pET33b(+) plasmid for expression of rRipE with 6x His-tags on both N- and C-terminus.
**pCis-FLAG-EHF0962-Gent-Himar A7 plasmid construction:**		
ripE	F: TG GGATCC ATAAGCACAAAAAACACTGAC<br>R: CA GGTACC TTATTGTGTTGATGAAAAATAC	Underlined: F, BamHI; R, KpnI.<br>*ripE* (start codon removed) was cloned into pCis-Himar A7 plasmid.
Gent (codon-optimized)	F: TG GGTACC ATTAAATTATGTTAAGATCATCAAATG<br>R: CA AAGCTT TTATGTTGCTGTACTTGG	Underlined: F, KpnI; R, HindIII.<br>Gentamicin resistance gene (*Gent*) was cloned into pCis-Himar A7 plasmid (Yan et al., 2018).
**qPCR and RT-qPCR primers:**		
Ehr16S	F: CGGGGGAAAGATTTATCGCTATTA<br>R: CGCTTGCCCCCTCCGTATTA	Specific qPCR primers for *Ehrlichia* 16S rRNA gene (Bekebrede et al., 2020)
mGAPDH	F: GTTGTCTCCTGCGACTTCA<br>R: GGTGGTCCAGGGTTTCTTA	Specific qPCR primers for mouse GAPDH gene (Bekebrede et al., 2020)
dGAPDH	F: ATCACTGCCACCCAGAAGAC<br>R: TCAGCTCAGGGATGACCTTG	Specific qPCR primers for dog GAPDH gene (Bekebrede et al., 2020)
ripE	F: CACAAGTACAGAATAGTGATGCTAC<br>R: AAATACTCCTTGAGTGAATGCCA	Specific qPCR primers for *E. japonica ripE* (*EHF_0962*)
**Gene-specific flank PCR primers:**		
H59 (*ripE*)	F: TCAATATAGAACAACATACTACAAA<br>R: TCTGCTTCATCAGATTCTACT	Primers flanking Himar1 insertion site in Δ*ripE* (Bekebrede et al., 2020)
mutS	F: GGAGACATTACGAGGTGTTGC<br>R: ACTGACCATGCAATTGACAATCC	Primers flanking Himar1 insertion site in rescued Δ*ripE* (Δ*ripE*$^{+}$)

EHF_0017	F: TAGGATGTGGTAACTTAGGAAGTA<br>R: ATCAATTCCAGCACACAAGG	Primers flanking Himar1 insertion site in *Eja* over-expressing RipE (WT^{+})
**Semi-random, two-step PCR (ST-PCR) primers for rescued Δ*ripE* and Eja+ over-expressing *ripE***		
Gent insertion site	F: TGGGTACCATTAAATTATGTTAAGATCATCAAATG<br>R: GGCCACGCGTCGACTAGTACNNNNNNNNNNGATAT	Step 1 of ST-PCR (Bekebrede et al., 2020)
Gent insertion site	F: GTTGGAGCTTTAGCAGCTTATGTATTACC<br>R: GGCCACGCGTCGACTAGTAC	Step 2 of ST-PCR (Bekebrede et al., 2020)
**Sequencing primers**		
T7T	GCTAGTTATTGCTCAGCGG	Sequencing primer for pET33b(+) plasmids
T7P	TAATACGACTCACTATAGGG	Sequencing primer for pET33b(+) plasmids
Gent (codon-optimized)	GTTGGAGCTTTAGCAGCTTATGTATTACC	Sequencing primer for Himar plasmid and *Ehrlichia* clones with Himar1 insertion

[1] *Eja*, *Ehrlichia japonica*.
[2] F, forward; R, reverse complement primers; underlined, restriction enzyme sites.

Table S2. Primary and secondary antibodies used in this study.

Target	Animal origin	Conjugation	Vendor/Source	Catalog #	Dilution	Applications
RipE	Mouse	–	This study	–	1 : 250<br>1 : 200	WB<br>IFA
Ehrlichia P28	Rabbit	–	(Ohashi et al., 1998)	–	1 : 1,000<br>1 : 300	WB<br>IFA
β-Actin (C4)	Mouse	–	Santa Cruz Biotech	sc-47778	1 : 500	WB
Ehrlichia HtrA	Rabbit	–	(Kumagai et al., 2010)	–	1 : 500	WB
Mouse IgA+IgG+IgM	Goat	HRP	SeraCare	5220-0342	1 : 2,000	WB
Rabbit IgG	Goat	HRP	SeraCare	5220-0336	1 : 2,000	WB
Mouse IgG	Goat	AF-488	Invitrogen	A11029	1 : 300	IFA
Rabbit IgG	Goat	AF-555	Invitrogen	A21429	1 : 300	IFA
Mouse IgG	Goat	AF-555	Invitrogen	A21424	1 : 300	IFA
Rabbit IgG	Goat	AF-488	Invitrogen	A11034	1 : 300	IFA

Abbreviations: RipE, resistance-inducing protein of *Ehrlichia*; WB, western blot; IFA, immunofluorescence assay; HRP, horseradish peroxidase; AF, Alexa Fluor.

Supplemental Figures

Figure S1. Growth curves of WT and ∆*ripE E. japonica* in RF/6A cultures had no significant difference.

Growth curves of WT- and ∆*ripE E. japonica*-infected RF/6A cells were analyzed by *Ehrlichia* 16S rRNA-specific qPCR. Input DNA was normalized by rhesus RPL32 gene. Data are displayed by the $2^{-\Delta\Delta CT}$ method and indicate the mean ± standard deviation (n = 3). The ratio of *Ehr16S*/RPL32 at 1 dpi was set as 1. Results were analyzed with repeated-measures ANOVA followed by Šídák multiple comparisons (P = 0.3898).

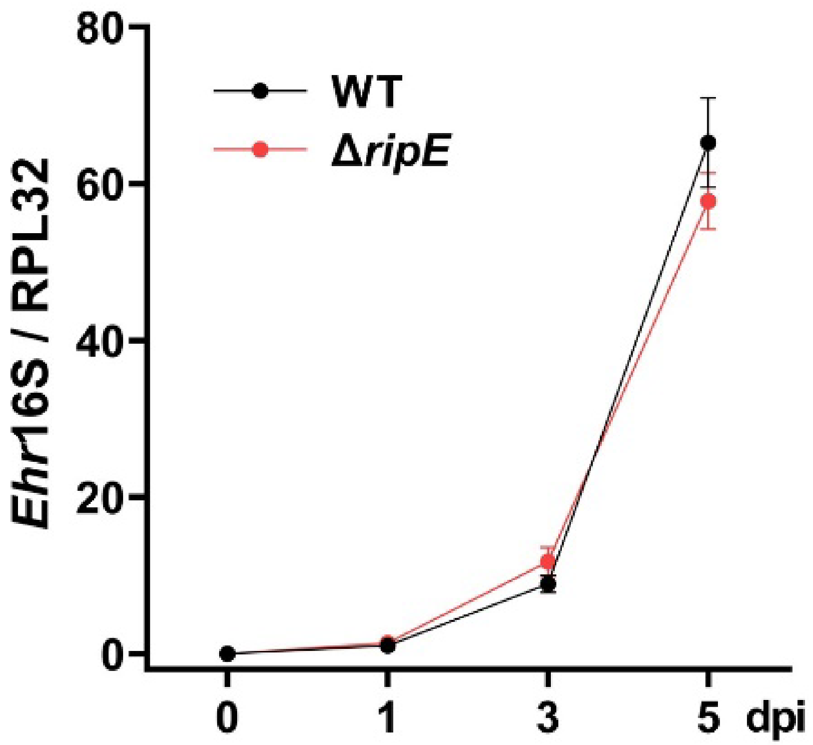

References:

Bekebrede, H., Lin, M., Teymournejad, O., and Rikihisa, Y. (2020). Discovery of in vivo Virulence Genes of Obligatory Intracellular Bacteria by Random Mutagenesis. *Front Cell Infect Microbiol* 10**,** 2. doi: 10.3389/fcimb.2020.00002.

Kumagai, Y., Matsuo, J., Hayakawa, Y., and Rikihisa, Y. (2010). Cyclic di-GMP signaling regulates invasion by *Ehrlichia chaffeensis* of human monocytes. *J Bacteriol* 192(16)**,** 4122-4133. doi: JB.00132-10 [pii] doi: 10.1128/JB.00132-10.

Ohashi, N., Zhi, N., Zhang, Y., and Rikihisa, Y. (1998). Immunodominant major outer membrane proteins of *Ehrlichia chaffeensis* are encoded by a polymorphic multigene family. *Infect. Immun.* 66(1)**,** 132-139.

Yan, Q., Lin, M., Huang, W., Teymournejad, O., Johnson, J.M., Hays, F.A., et al. (2018). Ehrlichia type IV secretion system effector Etf-2 binds to active RAB5 and delays endosome maturation. *Proc Natl Acad Sci U S A*. doi: 10.1073/pnas.1806904115.
